# Functional Imaging and Inhibitor Screening of Human Pancreatic Lipase by a Resorufin-Based Fluorescent Probe

**DOI:** 10.3390/bios13020283

**Published:** 2023-02-16

**Authors:** Fan-Bin Hou, Na Zhang, Guang-Hao Zhu, Yu-Fan Fan, Meng-Ru Sun, Liang-Liang Nie, Guang-Bo Ge, Yue-Juan Zheng, Ping Wang

**Affiliations:** 1 Shanghai Frontiers Science Center of TCM Chemical Biology, Institute of Interdisciplinary Integrative Medicine Research, Shanghai University of Traditional Chinese Medicine, Shanghai 201203, China; 2 Department of Biology, Philipps University, Karl-von-Frisch-Straße 8, 35043 Marburg, Germany; 3 The Research Center for Traditional Chinese Medicine, Shanghai Institute of Infectious Diseases and Biosecurity, Shanghai University of Traditional Chinese Medicine, Shanghai 201203, China; 4 School of Pharmaceutical Science, Liaoning University, Shenyang 110036, China

**Keywords:** human pancreatic lipase (hPL), high-throughput screening, fluorescence imaging, enzyme inhibitors

## Abstract

Human pancreatic lipase (hPL) is a crucial digestive enzyme responsible for the digestion of dietary lipids in humans, and inhibition of hPL is effective in reducing triglyceride intake, thereby preventing and treating obesity. In this study, a series of fatty acids with different carbon chain lengths were constructed to the fluorophore resorufin based on the substrate preference of hPL. Among them, RLE was found to have the best combination of stability, specificity, sensitivity and reactivity towards hPL. Under physiological conditions, RLE can be rapidly hydrolyzed by hPL and released to resorufin, which triggered approximately 100-fold fluorescence enhancement at 590 nm. RLE was successfully applied for sensing and imaging of endogenous PL in living systems with low cytotoxicity and high imaging resolution. Moreover, a visual high-throughput screening platform was established using RLE, and the inhibitory effects of hundreds of drugs and natural products toward hPL were evaluated. Collectively, this study reports a novel and highly specific enzyme-activatable fluorogenic substrate for hPL that could serve as a powerful tool for monitoring hPL activity in complex biological systems and showcases the potential to explore physiological functions and rapid screening of inhibitors.

## 1. Introduction

Human pancreatic lipase (hPL) is secreted by the pancreas and released into the gastrointestinal system, where it collaborates with bile salts secreted by the liver to break down fats into fatty acids and glycerol [1,2]. hPL plays an important role in the regulation of lipid metabolism, as it catalyzes the hydrolysis of approximately 50% to 70% of dietary fat [3]. Therefore, the activity of hPL is essential for lipid absorption, and inhibition of hPL is effective in reducing triglyceride intake, thereby preventing and treating obesity [4]. Obesity and overweight are closely associated with diabetes, hypertension, hyperlipidemia, fatty liver, cardiovascular disease and many other metabolic diseases [5,6,7]. Treating obesity can significantly reduce the prevalence of chronic metabolic diseases and mortality [8]. Orlistat is the only hPL inhibitor currently approved by the FDA, but its side effects of, such as diarrhea, bloating and fecal incontinence, are unfavorable, incidences as high as 91% [9,10,11]. Therefore, to address the health hazards caused by obesity, the priority is to develop safer and more effective drugs. In this context, rapid and effective screening methods are a prerequisite for drug development. In addition to its key role in lipid metabolism, hPL has been reported as a biomarker for a number of pancreatic diseases, such as chronic pancreatitis and pancreatic cancer [12,13]. This suggests that a reliable technique is now required for the quick, secure and sensitive monitoring of enzyme activity in cells, tissues and animals.

Current detection methods for hPL activity mainly rely on mass spectrometry or high-performance liquid chromatography (HPLC) [14,15]; however, owing to its high cost and cumbersome operational steps, it is not sufficient for real-time, in situ monitoring of target enzyme activity and high-throughput screening of inhibitors. Activity-based fluorescence substrate sensing represents a powerful detection strategy that relies on photochemistry and has been attracting increasing attention owing to its inherent advantages of low cost, simplicity of operation, suitability for high-throughput detection and provision of high spatiotemporal resolution [16,17,18,19]. Many studies have been devoted to the development of fluorescent substrates for pancreatic lipase (PL) (Table S1). However, most reported PL fluorescent substrates are subject to poor chemical stability, low selectivity and weak resistance to interference, which restrict their application [20,21,22,23,24]. In addition, most of the reported methods were used in combination with porcine pancreatic lipase (pPL) to replace hPL as the enzyme source [25,26]. Although pPL and hPL have high amino acid sequence identity (86%) and a conserved catalytic triad (Asp-His-Ser), species differences in inhibitor response towards these two lipases have been reported [27,28]. Hence, the study of the biological function of hPL and the high-throughput screening of inhibitors requires practical and reliable tools to detect the activity of hPL in complex biological systems.

It is well known that resorufin is frequently used in the construction of fluorescent substrates due to its low background fluorescence interference, stable light signal, strong resistance to photobleaching, good water solubility and biocompatibility [29,30]. Moreover, resorufin substrates are easy to obtain and very suitable for a wide range of applications. Therefore, in this study, we used resorufin as a fluorophore to design a novel and highly specific fluorescent substrate for the detection of hPL activity in complex biological systems and provide high-throughput screening of inhibitors. To achieve this, a series of resorufin esters were rationally designed to optimize the selectivity and sensitivity of the fluorescent substrate towards hPL. Among them, resorufin lauryl ester (RLE) displayed the best combination of stability, specificity, sensitivity and reactivity towards hPL. RLE was then successfully applied for sensing and imaging of endogenous hPL in living systems with low cytotoxicity and high imaging resolution. The RLE-based fluorescence assay enabled rapid and efficient screening of inhibitors and identified 6 excellent inhibitors of hPL among 94 drugs commonly used in clinical practice, as well as 94 natural products derived from Chinese medicine.

## 2. Materials and Methods

### 2.1. Materials and Instruments

Expression and purification of hPL were performed in accordance with previous studies conducted in our laboratory [31], and hCES1A and hCES2A were purchased from BD (Bergen, NJ, USA). CA, AchE, BchE, 5-HKE, lysozyme, trypsin, thrombin, α-CT, FAP, PREP, pepsin, α-amylase, plasmin, notum and hSA were purchased from Sigma-Aldrich (St. Louis, MO, USA). AR42J (cells from rat exocrine pancreas) was provided by ATCC (ATCC number: CRL-1492). All probes were dissolved in DMSO (LC grade, Tedia, OH, USA). Tris-HCl buffer (pH = 7.4, 25 mM, 150 mM NaCl, 1 mM CaCl_2_) was prepared with Mili-Q water (Millipore, MA, USA). Screened compounds were mainly obtained from Bidepharm (Shanghai, China), Chengdu Pfeiffer Biotechnology Co. (Shanghai, China) and Makclin Biochemical Co. (Shanghai, China). Stock solutions of each substrate or each tested inhibitor were dissolved in acetonitrile and stored at −20 °C until use. Some of the active ingredients of the herbal medicine were purchased from Tianjiang Pharmaceutical Co., Ltd. (Jiangyin, Jiangsu, China). All fluorescence intensity measurements were recorded using a multimode enzyme spectrometer (SpectraMax iD3, Molecular Devices, San Jose, CA, USA). All other reagents and solvents were of the highest commercially available grade. The structure of the synthesized compounds before dissolution in DMSO-*d*_6_ or CDCl_3_ (Euriso-Top, Saint Aubin, France) was determined by a Bruker 600 NMR. Chemical shifts were recorded by ^1^H-NMR (400 MHz or 600 MHz) and ^13^C-NMR (101 MHz or 151 MHz).

### 2.2. Chemistry

**General procedure for ester derivatives of Resorufin**. Acyl chloride was added dropwise (1.5 equiv) to a solution of resorufin and Et_3_N (4.0 equiv) in DMF in an ice bath. Then, the reaction was warmed to room temperature and stirred for 4 h. After completion of the reaction as indicated by TLC monitoring, water was added and extracted with ethyl acetate. The combined organic layers were washed with brine, dried over Na_2_SO_4_, filtered and concentrated. The residue was purified by silica gel flash column chromatography to afford the desired compound.

***3-oxo-3H-phenoxazin-7-yl butyrate* (Resorufin butyryl ester)** Compound **Resorufin butyryl ester** was prepared from butyl chloride according to the following general procedure. Yield: 79.2%. Yellow solid. ^1^H NMR (400 MHz, CDCl_3_) δ 7.82 (d, *J* = 8.6 Hz, 1H), 7.46 (d, *J* = 9.8 Hz, 1H), 7.21–7.12 (m, 2H), 6.89 (dd, *J* = 9.8, 2.0 Hz, 1H), 6.35 (d, *J* = 2.0 Hz, 1H), 2.62 (t, *J* = 7.4 Hz, 2H), 1.83 (h, *J* = 7.4 Hz, 2H), 1.09 (t, *J* = 7.4 Hz, 3H). ^13^C NMR (101 MHz, CDCl_3_) δ 186.34, 171.27, 153.64, 149.35, 148.24, 144.38, 135.16, 134.81, 131.18, 131.14, 119.33, 109.71, 107.23, 36.19, 18.32, 13.60. ESI-MS: M = 283.08; found m/z 284.09 [M + H]^+^.

***3-oxo-3H-phenoxazin-7-yl hexanoate* (Resorufin hexanoyl ester)** Compound **Resorufin hexanoyl ester** was prepared from hexanoyl chloride according to the following general procedure. Yield: 80.2%. Yellow solid. ^1^H NMR (400 MHz, CDCl_3_) δ 7.82 (d, *J* = 8.6 Hz, 1H), 7.46 (d, *J* = 9.8 Hz, 1H), 7.21–7.12 (m, 2H), 6.89 (dd, *J* = 9.8, 2.0 Hz, 1H), 6.35 (d, *J* = 2.1 Hz, 1H), 2.63 (t, *J* = 7.5 Hz, 2H), 1.87–1.74 (m, 2H), 1.50–1.37 (m, 4H), 1.02–0.91 (m, 3H). ^13^C NMR (101 MHz, CDCl_3_) δ 186.34, 171.46, 153.66, 149.36, 148.23, 144.38, 135.16, 134.81, 131.18, 131.13, 119.32, 109.71, 107.23, 34.35, 31.21, 24.47, 22.30, 13.91. ESI-MS: M = 311.12; found m/z 312.12 [M + H]^+^.

***3-oxo-3H-phenoxazin-7-yl octanoate* (Resorufin octanoyl ester)** Compound **Resorufin octanoyl ester** was prepared from octanoyl chloride according to the following general procedure. Yield: 80.5%. Yellow solid. ^1^H NMR (400 MHz, CDCl_3_) δ 7.82 (d, *J* = 8.6 Hz, 1H), 7.46 (d, *J* = 9.7 Hz, 1H), 7.22–7.11 (m, 2H), 6.89 (dd, *J* = 9.9, 2.0 Hz, 1H), 6.35 (d, *J* = 2.0 Hz, 1H), 2.62 (t, *J* = 7.5 Hz, 2H), 1.79 (p, *J* = 7.4 Hz, 2H), 1.50–1.25 (m, 8H), 1.05–0.89 (m, 3H). ^13^C NMR (101 MHz, CDCl_3_) δ 186.35, 171.46, 153.67, 149.36, 148.23, 144.38, 135.16, 134.81, 131.18, 131.13, 119.32, 109.71, 107.23, 34.38, 31.63, 29.02, 28.89, 24.79, 22.60, 14.07. ESI-MS: M = 339.15; found m/z 340.15 [M + H]^+^.

***3-oxo-3H-phenoxazin-7-yl decanoate* (Resorufin decanoyl ester)** Compound **Resorufin decanoyl ester** was prepared from decanoyl chloride according to the following general procedure. Yield: 80.5%. Yellow solid. ^1^H NMR (600 MHz, CDCl_3_) δ 7.82 (d, *J* = 8.7 Hz, 1H), 7.46 (d, *J* = 9.8 Hz, 1H), 7.20–7.11 (m, 2H), 6.89 (dd, *J* = 9.8, 2.0 Hz, 1H), 6.35 (d, *J* = 2.0 Hz, 1H), 2.62 (t, *J* = 7.5 Hz, 2H), 1.79 (*p*, *J* = 7.5 Hz, 2H), 1.47–1.30 (m, 12H), 0.91 (t, *J* = 6.8 Hz, 3H). ^13^C NMR (151 MHz, CDCl_3_) δ 186.33, 171.46, 153.67, 149.36, 148.24, 144.38, 135.16, 134.81, 131.18, 131.13, 119.32, 109.71, 107.23, 34.39, 31.86, 29.40, 29.25, 29.23, 29.08, 29.06, 24.80, 22.67, 14.11. ESI-MS: M = 367.18; found m/z 368.19 [M + H]^+^.

***3-oxo-3H-phenoxazin-7-yl dodecanoate* (RLE)** Compound **RLE** was prepared from lauroyl chloride according to the following general procedure. Yield: 82.5%. Yellow solid. ^1^H NMR (400 MHz, CDCl_3_) δ 7.82 (d, *J* = 8.6 Hz, 1H), 7.46 (d, *J* = 9.8 Hz, 1H), 7.21–7.12 (m, 2H), 6.89 (dd, *J* = 9.8, 2.0 Hz, 1H), 6.36 (d, *J* = 2.0 Hz, 1H), 2.62 (t, *J* = 7.5 Hz, 2H), 1.82–1.76 (m, 2H), 1.29 (d, *J* = 5.9 Hz, 17H), 0.89 (d, *J* = 7.0 Hz, 3H). ^13^C NMR (101 MHz, CDCl_3_) δ 186.37, 171.48, 153.68, 149.37, 148.22, 144.38, 135.15, 134.82, 131.18, 131.14, 119.34, 109.71, 107.22, 34.38, 33.65, 31.91, 29.60, 29.44, 29.34, 29.25, 29.23, 29.08, 29.06, 24.79, 24.74, 22.69, 14.12. ESI-MS: M = 395.21; found m/z 396.22 [M + H]^+^.

***3-oxo-3H-phenoxazin-7-yl palmitate* (Resorufin palmitoyl ester)** Compound **Resorufin palmitoyl ester** was prepared from palmitoyl chloride according to the following general procedure. Yield: 83.2%. Yellow solid. ^1^H NMR (400 MHz, CDCl_3_) δ 7.82 (d, *J* = 8.6 Hz, 1H), 7.46 (d, *J* = 9.8 Hz, 1H), 7.20–7.11 (m, 2H), 6.89 (dd, *J* = 9.8, 2.0 Hz, 1H), 6.36 (d, *J* = 2.0 Hz, 1H), 2.62 (t, *J* = 7.5 Hz, 2H), 1.78 (q, *J* = 7.5 Hz, 2H), 1.45–1.28 (m, 24H), 0.90 (t, *J* = 6.7 Hz, 3H). ^13^C NMR (101 MHz, CDCl_3_) δ 186.35, 171.47, 153.67, 149.36, 148.22, 144.38, 135.16, 134.82, 131.18, 131.13, 119.33, 109.71, 107.23, 34.39, 31.93, 29.70, 29.66, 29.65, 29.59, 29.44, 29.37, 29.23, 29.09, 29.06, 24.79, 22.70, 14.13. ESI-MS: M = 451.27; found m/z 452.39 [M + H]^+^.

***3-oxo-3H-phenoxazin-7-yl stearate* (Resorufin stearyl esters)** Compound. **Resorufin stearyl ester** was prepared from stearoyl chloride according to the following general procedure. Yield: 87.3%. Yellow solid. ^1^H NMR (600 MHz, CDCl_3_) δ 7.82 (d, *J* = 8.6 Hz, 1H), 7.46 (d, *J* = 9.7 Hz, 1H), 7.19–7.12 (m, 2H), 6.89 (d, *J* = 9.8 Hz, 1H), 6.35 (s, 1H), 2.62 (t, *J* = 7.5 Hz, 2H), 1.79 (*p*, *J* = 7.5 Hz, 2H), 1.47–1.27 (m, 28H), 0.90 (t, *J* = 6.9 Hz, 3H). ^13^C NMR (151 MHz, CDCl_3_) δ 186.32, 171.46, 153.67, 149.35, 148.25, 144.38, 135.16, 134.81, 131.18, 131.13, 119.32, 109.71, 107.24, 34.39, 31.93, 29.71, 29.69, 29.67, 29.65, 29.60, 29.45, 29.37, 29.23, 29.07, 24.80, 22.70, 14.13. ESI-MS: M = 479.30; found m/z 480.31 [M + H]^+^.

### 2.3. Selectivity and Sensitivity of RLE to hPL

RLE was incubated with hPL and various enzymes, such as hCES1A, hCES2A, CA, AchE, BchE, 5-HKE, lysozyme, trypsin, thrombin, α-CT, FAP, PREP, pepsin, α-amylase, plasmin, notum and hSA, in a 100 µL incubation mixture consisting of Tris-HCl (pH = 7.4, 25 mM), NaCl (150 mM), CaCl_2_ (1 mM) and porcine bile salt (0.1 mg/mL) at a concentration of 1.5 µg/mL. The reaction was initiated by adding RLE to the preincubated enzyme mixture at 37 °C, and metabolite formation was determined by measuring the fluorescence intensity of resorufin at 590 nm. In addition, several amino acids (IIe, His, Trp, Val, Leu, Tyr, Ala, Thr, Pro, Asp, Lys, Met, Cys, Ser, Phe, Gly, Glu and Gln), ions (Ni^2+^, Ca^2+^, Na^+^, K^+^, Cu^2+^, Zn^2+^, Mg^2+^, NH_4_^+^, Cu^+^, Fe^2+^ and CN^-^) and GSH were measured. The concentration of these interfering factors was 5 μM.

### 2.4. Enzymatic Kinetics of hPL-Mediated RLE Hydrolysis

A kinetic study was carried out to estimate the kinetic parameters of **RLE** hydrolysis in hPL. **RLE** (previously dissolved in DMSO) was serially diluted to the desired concentrations (1, 2.5, 4.0, 7.5, 10.0, 12.0 and 15.0 µM) and mixed with hPL solution (1.5 µg/mL final concentration) in which the bile salt concentration was 0.1 mg/mL. The buffer was Tris-HCl. The change in the fluorescence spectrum was then measured at 590 nm. Data analysis was performed by nonlinear regression using GraphPad Prism 6 (GraphPad Software, San Diego, CA, USA).

$$V=\frac{Vmax\times \left[S\right]}{Km+\left[S\right]}$$

### 2.5. Cell Viability Assay

AR42J cells were inoculated at a density of 1 × 10^4^ in 200 μL in Dulbecco’s modified Eagle medium containing 10% FBS and maintained in a 37 °C, 5% CO_2_ incubator for 24 h. Cells were then incubated with different concentrations of RLE (0, 0.1, 0.5, 1, 5, 10, 20, 30, 40 and 50 μM) for 48 h, washed once with 37 °C PBS and incubated for a further 3 h with the addition of medium containing 10% CCK-8. Absorbance was measured at 450 nm.

### 2.6. Confocal Microscopic Imaging of PL in AR42J Cells

AR42J cells were cultured overnight in DMEM containing 10% fetal bovine serum (FBS) at 37 °C in 5% CO_2_, followed by coculture with 0.5 μM caerulein in FBS-free medium for 24 h [32]. After washing twice with medium, the cells were incubated with/without 25 μM orlistat (prepared in FBS-free medium) in a 5% CO_2_ incubator for 30 min at 37 °C. RLE stock solution (20 mM) was diluted with FBS-free cell culture medium to a final concentration of 10 μM, and cells were then incubated with FBS-free cell culture medium containing RLE for a further 30 min at 37 °C. After washing three times with PBS, the cells were imaged under a confocal fluorescence microscope (Leica SP8, Wetzlar, Germany): blue channel, *λ_ex_* = 405 nm, *λ_em_* = 415–485 nm; red channel, *λ_ex_* = 552 nm, *λ_em_* = 580–600 nm.

### 2.7. Fluorescence Imaging of Monkey Pancreas Slices

Slices were prepared from a cynomolgus pancreas obtained from a 4-year-old male cynomolgus monkey from JOINN Lab (Suzhou, China). Monkey pancreas tissue was sliced to a thickness of 150 μm using a Microtome cryostat (Leica, Wetzlar, Germany) and placed in dish (Ø 20 mm). For the blank group, neither RLE nor inhibitors were added. For the experimental group, sections were incubated with PBS containing RLE (15 μM) for 30 min only. For the inhibitor group, sections were treated with orlistat (25 μM) for 15 min, then incubated with RLE (15 μM) for 30 min. Finally, the samples were examined with a confocal microscope (Leica SP8, Wetzlar, Germany); *λ_ex_* = 552 nm, *λ_em_* = 580–600 nm.

### 2.8. Molecular Docking Simulations

Docking simulations were performed using AutoDock Vina (1.2.3) to analyze the binding between RLE and hPL. hPL (PDB:1lpa) and RLE structures were preprocessed by AutoDockTools-1.5.7. Docking sites for RLE were defined as described in the literature [33]. The RLE was then docked to the active site, and the docking poses were scored by Vina 1.2.3. The apo-hPL or the hPL-RLE complex structure was solvated in a dodecahedron water box of TIP3 water molecules with a spacing of 1 nm in all directions from the walls of the box. The sodium chloride concentration was set to 0.15 M. The model systems were first energy-minimized with the heavy atoms of protein and ligand using the steepest descent algorithm with a maximum 50,000 steps until the maximum force < 10.0 kJ/mol. The temperature was equilibrated to 310 K in the NVT ensemble for 1 ns (V-rescale thermostat), and then the model systems were equilibrated in the NPT ensemble at 1 bar for 1 ns (Parrinello–Rahman barostat) [34,35]. In the production phase, the temperature and pressure were kept at 310 K and 1 bar, respectively using a V-rescale thermostat and a Parrinello–Rahman barostat. All the model systems were implemented in 100 ns MD simulations. Trajectories of simulations were further analyzed and visualized by PyMOL (The PyMOL Molecular Graphics System 2.4.0a0 Open-Source, Schrödinger LLC., New York, NY, USA) and Discovery Studio Visualizer (BIOVIA Discovery Studio 2019, Dassault Systèmes, San Diego, CA, USA).

### 2.9. hPL Inhibition Assay

An RLE-based fluorescence assay was used to measure the residual activity of hPL in order to determine the inhibitory potential of various compounds on hPL, and orlistat was selected as a positive inhibitor. First, we evaluated the inhibitory effect of 94 natural products derived from herbs and 94 drugs on hPL. In our high-throughput screening system, 1 ug/mL hPL was used, and the final concentration of the 94 natural products derived from herbs and 94 drugs used was 10 μM. Other conditions were the same as in the selectivity experiments. Additionally, IC_50_ values were determined for the compounds exhibiting the strong inhibitory activity for hPL.

## 3. Results and Discussion

### 3.1. Design, Synthesis and Sensing Mechanisms of RLE

**Figure 1:**
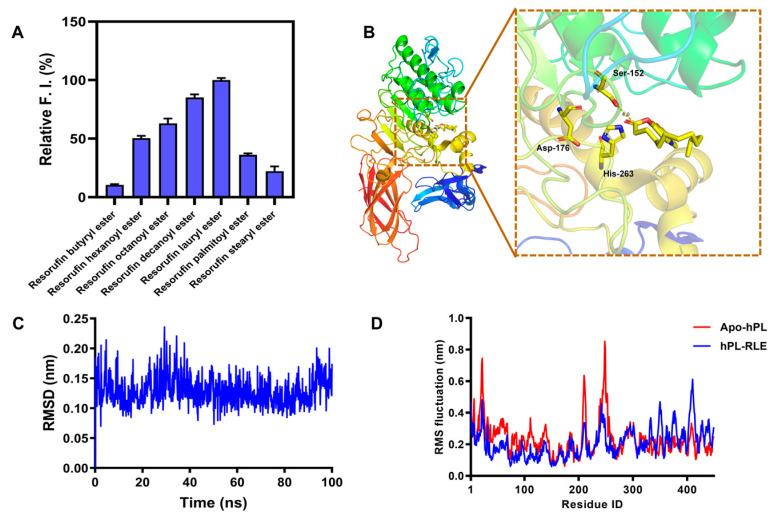
(**A**) Relative fluorescence intensity of each designed substrate (5 µM) towards hPL at 37 °C. (**B**) Docking molecular interaction between hPL and the RLE probe. (**C**) RMSD distribution of RLE on hPL lipase. (**D**) RMSF distribution of residues of hPL lipase and Apo-hPL lipase, as well as hPL binding with RLE. All data are expressed as mean ± SD.

**Figure 2:**
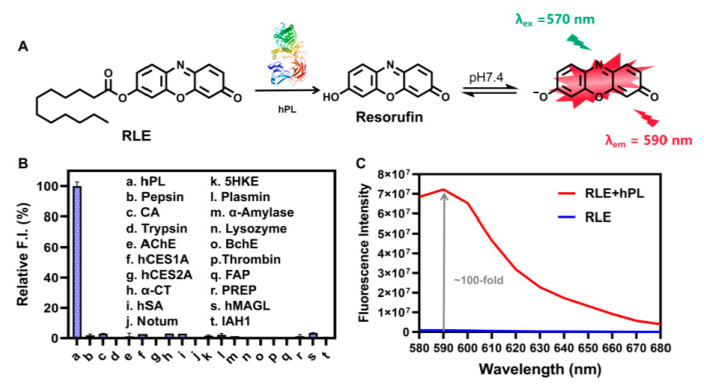
(**A**) Proposed sensing mechanism of the hPL-triggered RLE fluorescence response. (**B**) Fluorescence response intensity of RLE with various hydrolase incorporations. (**C**) Fluorescence emission spectra of RLE (5 μM) in the presence and absence of hPL; *λ_em_* = 590 nm. All data are expressed as mean ± SD.

**Scheme 1:**
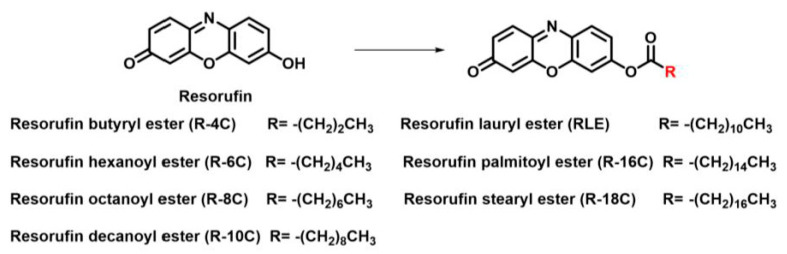
Synthesis of resorufin ester derivatives.

Enzyme recognition moiety is an indispensable element for fluorogenic substrates that is extremely responsible for selective and sensitive interactions with the target enzyme of interest [36]. Herein, based on the substrate preference of hPL, a series of fatty acids with different carbon chain lengths was constructed to the fluorophore resorufin as the recognition moiety of hPL. Mechanistically, esterification of the hydroxyl group of resorufin reduces its electron donor ability and inhibits the intramolecular charge transfer (ICT) process. When the ester group was selectively recognized by the hPL and cleaved in the enzyme-catalyzed reaction, then fluorophore resorufin was released to yield fluorescence due to the recovery of ICT progress. The chemical structures of the six designed resorufin esters are depicted in Scheme 1. Experimental details of the synthesis and structural characterization of the six ester compounds are described in the Supplementary Information. The screening results show that the resorufin lauryl ester (RLE) had the best reactivity towards hPL (Figure 1A), the molecular docking results show that RLE had a good affinity for hPL (−8.0 kcal/mol) and the distance between the carbonyl carbon of RLE and the hydroxyl oxygen of ser-152 of the hPL was extremely short (only 4.0 Å) (Figure 1B). During the 100 ns molecular dynamics simulations, the RMSD distribution of RLE vibrated to a very limited extent, suggesting that RLE stably bound to the catalytic pocket of hPL (Figure 1C). Furthermore, RMS fluctuation of the flap region (residues 237–261) was also dramatically steadied after combination with RLE (Figure 1D), the removal and reorientation of which by the substrate resulted in the catalytic activation of hPL. The sensibility of the RLE towards hPL was then carefully investigated. RLE was stable in buffer (Tris-HCl, pH 7.4) at 37 °C. After co-incubation with hPL, RLE (Φ = 0.007) was rapidly hydrolyzed and released resorufin (Φ = 0.19, *λ_ex_* = 570 nm), with a significant increase in fluorescence at 590 nm (Figure S1). High-performance liquid chromatography (HPLC) and high-resolution mass spectrometry (HRMS) demonstrated that the change in fluorescence correlated with the production of resorufin (Figure S2). The mechanism of hPL sensing by RLE is described in Figure 2A.

### 3.2. Selectivity and Sensitivity of RLE towards hPL

Since substrate specificity is a vital property affecting the performance of activity-based fluorogenic substrate analysis, we further scrutinized the specificity of the RLE for hPL under physiological conditions [37]. As shown in Figure 2B, only hPL caused a significant fluorescence enhancement at 590 nm, while there was no noticeable change with other enzymes, including carboxylesterases (hCES1A and hCES2A), cholinesterases (AChE and BChE), carbonic anhydrase (CA), pepsin, trypsin and peptidase (FAP), as well as human serum albumin (hSA), which means that RLE has a good selectivity towards hPL. RLE was rapidly hydrolyzed by hPL and released to resorufin, which triggered approximately 100-fold fluorescence enhancement at 590 nm (Figure 2C). Furthermore, the photostability and chemical stability of RLE and its hydrolysis product, resorufin, were also examined (Figures S3–S5). The fluorescence intensity of neither RLE nor resorufin changed markedly after 60 min of irradiation with a 550 nm xenon lamp, indicating that RLE and resorufin have outstanding photostability. The fluorescence intensity of RLE is extremely weak over a wide pH range, whereas resorufin fluoresces strongly in the pH range of 6.5–10.5. These results indicate that RLE and resorufin have favorable chemical stability. Various interfering factors, such as various amino acids (Cys, Ser, Lys, etc.), organic ions (Fe^2+^, Cu^+^, CN^−^, etc.) and GSH, do not affect the fluorescence response of RLE towards hPL. These observations indicate that RLE could serve as a highly desirable fluorogenic substrate for sensing hPL based on its unique enzymatic activity.

### 3.3. Enzymatic Kinetics of RLE Hydrolysis

**Figure 3:**
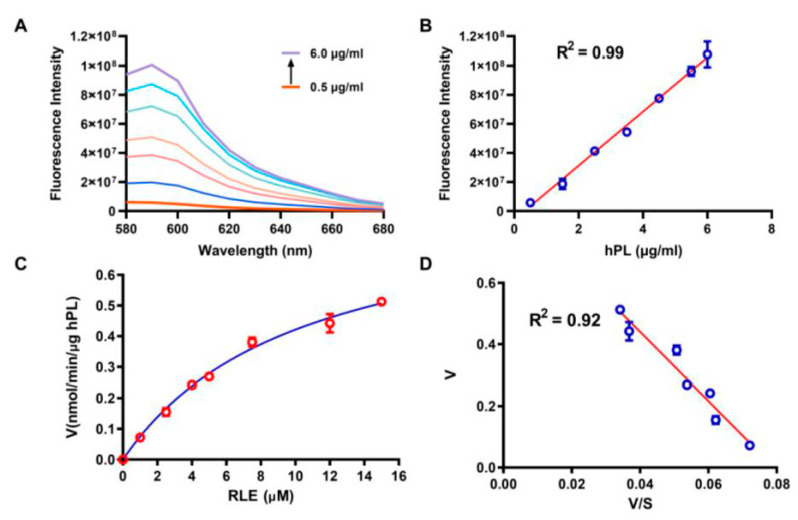
(**A**) Fluorescence spectra of RLE (5 μM) incubated with increasing concentrations of hPL (0.5–6 µg/mL) in Tris-HCl. (**B**) The linear relationship between fluorescence intensity and hPL concentrations (0.5–6 µg/mL) in Tris-HCl. (**C**) Hydrolytic kinetics of RLE (5 μM) in hPL at 37 °C. (**D**) Eadie–Hofstee plot. All data are expressed as mean ± SD.

Enzymatic kinetic behavior is another key property not only for quantitative applications of activity-based fluorogenic substrates but also for the further identification of enzyme inhibitors [38]. To this end, the linear range of the fluorescence response of RLE towards hPL was evaluated preliminary. As shown in Figure 3A,B, the fluorescence intensity increased with increasing enzyme concentration, with good linearity in the enzyme concentration range of 0.5–6.0 µg/mL. The detection limit of RLE for hPL was determined to be 0.369 µg/mL. The time fluorescence responses of RLE towards hPL also exhibited good linearity with an incubation time up to 10 min (Figure S6). After that, the hydrolytic kinetics of RLE were carefully characterized using hPL as the enzyme source. As shown in Figure 3C,D, the hydrolytic behavior of RLE in hPL was consistent with classical Michaelis–Menten kinetics, and the corresponding Eadie–Hofstee plots provided evidence for this conclusion. RLE showed high affinity and good responsiveness to hPL (*K_m_* = 10.36 µM; *V_max_* = 0.86 nmol/min/µg/hPL). The great kinetic parameters and properties of the probe provided the basis for its further application to the detection of complex biological samples.

### 3.4. Confocal Microscopic Imaging of PL in Living Cells and Tissue Slices

**Figure 4:**
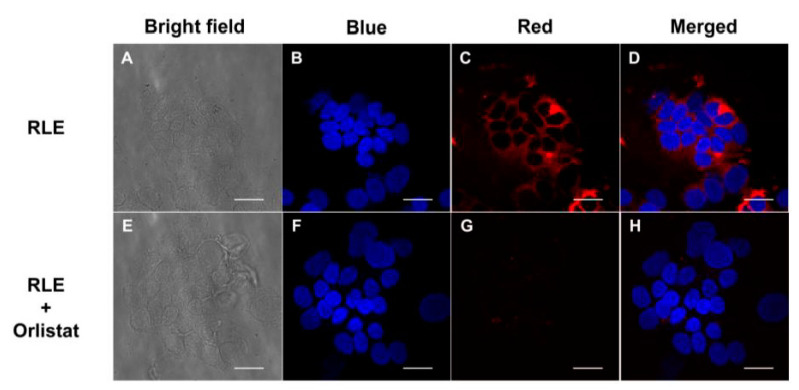
Imaging in live AR42J cells. (**A**–**D**) AR42J cells incubated with RLE (15 μM) and Hoechst 33,342 (5 μg/mL) for 20 min. (**E**–**H**) Colocalization images of live AR42J cells costained with RLE (15 μM) after treatment with orlistat (25 μM) for 15 min. Scale bars = 25 μm. Blue channel for Hoechst 33,342, 420–480 nm; red channel for RLE, 580–600 nm.

High specificity and excellent fluorescence properties enabled the probe to bio-image the activity of endogenous PL in living cells and tissues. Prior to performing cellular imaging, the cytotoxicity of RLE was evaluated by a cholecystokinin octapeptide (CCK-8) assay. As shown in Figure S7, cell viability remained above 80% after 48 h of incubation at high concentrations of RLE (50 μM), which means that the RLE has great biocompatibility. Subsequently, AR42J cells were treated with RLE (20 µM) at 37 °C for 30 min, and a marked fluorescent signal was observed in the red channel (Figure 4C). On the contrary, the fluorescent signal was significantly reduced after the addition of a specific PL inhibitor, orlistat (Figure 4G and Figure S9). Furthermore, the specificity of RLE towards PL in AR42J cell preparations was investigated. The results indicate that RLE can be rapidly hydrolyzed and released resorufin in AR42J cells preparations, whereas hydrolysis of RLE can be significantly blocked by orlistat (Figure S10). These results substantiate that RLE can be used for the sensing of endogenous PL in living cells.

**Figure 5:**
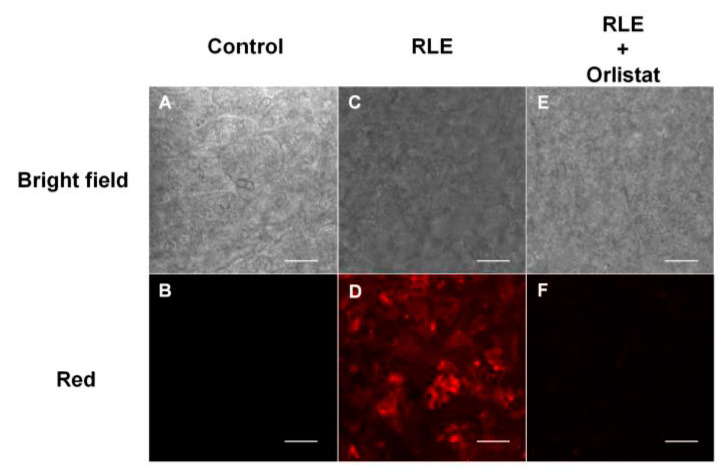
Confocal images of monkey pancreas slices. (**A**,**B**) Slices incubated with PBS only for 30 min. (**C**,**D**) Slices incubated with RLE (15 μM) for 20 min. (**E**,**F**) Slices incubated with RLE (15 μM) after treatment with orlistat (25 μM) for 15 min. Red channel for RLE: *λ_ex_* = 552 nm, *λ_em_* = 580 − 600 nm. Scale bars = 25 μm.

Encouraged by the excellent detection capability of this probe, we further evaluated the ability of RLE to detect endogenous PL in monkey pancreatic tissues (Figure 5). Similar to the cell imaging results, red-channel fluorescence was clearly captured in tissue sections after coincubation with RLE (15 µM) for 30 min (Figure 5D), while the fluorescent signal was significantly diminished following pretreatment of orlistat (Figure 5F and Figure S11). In addition, the hydrolysis of RLE in monkey pancreatic tissue preparations was also significantly inhibited by orlistat (Figure S12). These results demonstrate that RLE is a credible and useful molecular tool for in situ sensing and imaging of endogenous PL in living cells and tissue slices with good cell permeability, low cytotoxicity and high imaging resolution.

### 3.5. High-Throughput Screening of hPL Inhibitors Based on RLE

**Figure 6:**
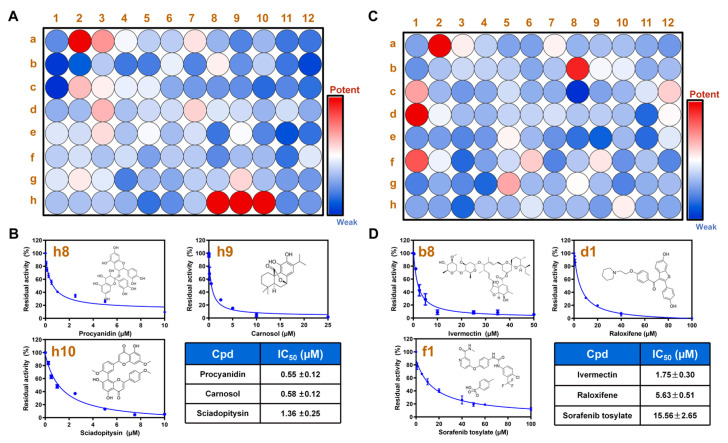
High-throughput screening of hPL inhibitors by RLE-based fluorescent assay. (**A**) Heat map for high-throughput screening of 94 natural products derived from herbs upon hPL inhibition. (**B**) Dose–inhibition curves of procyanidin (h8), carnosol (h9), and sciadopitysin (h10) against hPL-catalyzed RLE hydrolysis. (**C**) Heat map for high-throughput screening of 94 drugs upon hPL inhibition. (**D**) Dose–inhibition curves of ivermectin (b8), raloxifene (d1) and sorafenib tosylate (f1) against hPL-catalyzed RLE hydrolysis.

The superior anti-interference properties and sensitivity prompted us to establish a high-throughput screening method for hPL inhibitors to identify potential hPL inhibitors as antiobesity agents. In this case, a visual high-throughput assay was established to screen hPL inhibitors, using RLE as a fluorogenic substrate in a 96-well microplate. As shown in Figure S8, orlistat, a specific PL inhibitor, strongly inhibited hPL-catalyzed RLE hydrolysis in a dose-dependent manner, with an IC_50_ value of 2.51 nM, which is consistent with its reported inhibitory activity (IC_50_ = 6.16 nM) [39]. Subsequently, the inhibitory effect of 94 drugs commonly used in clinical practice and 94 natural products derived from Chinese medicine was determined (Figure 6A,C). The heat map clearly shows that six strong inhibitors stood out with a remarkable hPL-inhibitory effect (Figure 6A: 8h procyanidin, 9h carnosol, 10h sciadopitysin; Figure 6B: 8b ivermectin, 1d Raloxifene and 1F sorafenib tosylate). As shown in Figure 6B,D, the IC_50_ values for procyanidin, carnosol and sciadopitysin were 0.55 µM, 0.58 µM and 1.36 µM, respectively; the IC_50_ values for ivermectin, raloxifene and sorafenib tosylate were 1.75 µM, 5.63 µM and 15.56 µM, respectively. These results demonstrate that the RLE-based assay holds great promise for visual high-throughput screening of hPL inhibitors.

## 4. Conclusions

In summary, a novel and highly selective fluorescent probe (RLE) towards hPL was rationally designed based on the substrate preference of hPL. Under physiological conditions, RLE can be rapidly hydrolyzed by hPL and released to resorufin, which triggered approximately 100-fold fluorescence enhancement at 590 nm. The proposed RLE-based fluorescence assay offers a novel, practical and reliable tool for quantitative detection of PL activity in complex biospecimens and high-throughput screening of PL inhibitors, in addition to in situ sensing of endogenous PL activity in living cells and tissues, with high spatiotemporal resolution.

## Data Availability

Data will be made available upon request.

## Supplementary Materials

The following supporting information can be downloaded at: https://www.mdpi.com/article/10.3390/bios13020283/s1. Figure S1: The change in absorption spectrum of RLE (20 µM) in presence of hPL (10 µg/mL); Figure S2: (A) Representative LC-UV chromatograms of RLE incubation samples at 37 °C, UV detector was set at 440 nm; Figure S3: The effects of pH values on the fluorescence intensity of RLE and its metabolite Resorufin (5 µM), PMT Gain = 500 volts; Figure S4: The photostability of RLE (5 µM) and Resorufin (5 µM), following continuous illumination at 550 nm for different time, PMT Gain = 500 volts; Figure S5: Fluorescence responses of RLE (5 µM) to various analytes in aqueous solution. Relative fluorescence intensity (%) = Fluorescence intensity of adding analytes / Fluorescence intensity of hPL; Figure S6: The Linear in fluorescence intensity of RLE (5 µM) over time upon addition of hPL (1 µg/mL) in buffer at 37 °C: Figure S7: The cytotoxicity of RLE in AR42J; Figure S8: Dose-inhibition curves of orlistat against hPL catalysed RLE hydrolysis. All data were expressed as mean ± SD; Figure S9: Graphical quantification of average fluorescence intensity of PL activities by RLE in AR42J cells; Figure S10: Inhibitory effect of orlistat (25 µM) on PL catalyzed RLE hydrolysis in AR42J cells S9; Figure S11: Graphical quantification of average fluorescence intensity of PL activities by RLE in Monkey pancreatic tissues; Figure S12: Inhibitory effect of orlistat (25 µM) on PL catalyzed RLE hydrolysis in monkey pancreas S9; Figure S13: ^1^H NMR spectra of compound Resorufin butyryl ester; Figure S14: ^13^C NMR spectra of compound Resorufin butyryl ester; Figure S15: HRMS spectrum of compound Resorufin butyryl ester; Figure S16: ^1^H NMR spectra of compound Resorufin hexanoyl ester; Figure S17: ^13^C NMR spectra of compound Resorufin hexanoyl ester; Figure S18: HRMS spectrum of compound Resorufin hexanoyl ester; Figure S19: ^1^H NMR spectra of compound Resorufin octanoyl ester; Figure S20: ^13^C NMR spectra of compound Resorufin octanoyl ester; Figure S21: HRMS spectrum of compound Resorufin octanoyl ester; Figure S22: ^1^H NMR spectra of compound Resorufin decanoyl ester; Figure S23: ^13^C NMR spectra of compound Resorufin decanoyl ester; Figure S24: HRMS spectrum of compound Resorufin decanoyl ester; Figure S25: ^1^H NMR spectra of compound RLE; Figure S26: ^13^C NMR spectra of compound RLE; Figure S27: ^1^H NMR spectra of compound Resorufin palmitoyl ester; Figure S28: ^13^C NMR spectra of compound Resorufin palmitoyl ester; Figure S29: HRMS spectrum of compound Resorufin palmitoyl ester; Figure S30: ^1^H NMR spectra of compound Resorufin stearyl esters; Figure S31: ^13^C NMR spectra of compound Resorufin stearyl esters; Figure S32: HRMS spectrum of compound Resorufin stearyl esters; Table S1: The reported fluorescent substrates for sensing pancreatic lipase; Table S2: Residual activity of 94 natural products derived from herbs (10 μM, final concentration) against hPL-catalyzed RLE hydrolysis; Table S3: Residual activity of 94 drugs (10 μM, final concentration) against hPL-catalyzed RLE hydrolysis.
