# Supplementary material for: Functional Imaging and Inhibitor Screening of Human Pancreatic Lipase by a Resorufin-Based Fluorescent Probe
Source: Biosensors (Basel). 2023 Feb 16;13(2):283. doi: 10.3390/bios13020283 (PMC9953885; doi:10.3390/bios13020283)
Supplement: Supplementary file 1 [file biosensors-13-00283-s001.zip › biosensors-2174901-supplementary.pdf]

Supplementary Materials

for

Functional imaging and inhibitor screening of human pancreatic lipase by a resorufin-based fluorescent probe

Fan-Bin Hou[1,†], Na Zhang[2,†], Guang-Hao Zhu[1], Yu-Fan Fan[1], Meng-Ru Sun[1], Liang-Liang Nie[3,4], Guang-Bo Ge[1], Yue-Juan Zheng[3,*], Ping Wang[1,*]

[1] Shanghai Frontiers Science Center of TCM Chemical Biology; Institute of Interdisciplinary Integrative Medicine Research, Shanghai University of Traditional Chinese Medicine, Shanghai, 201203, China
[2] Department of Biology, Philipps University, Karl-von-Frisch-Straße 8, Marburg, 35043, Germany
[3] The Research Center for Traditional Chinese Medicine, Shanghai Institute of Infectious Diseases and Biosecurity, Shanghai University of Traditional Chinese Medicine, Shanghai, 201203, China
[4] School of Pharmaceutical Science, Liaoning University, Shenyang 110036, China

* Correspondence: pwang@shutcm.edu.cn (P.W.), zhengyj@shutcm.edu.cn (Y.-J. Z.)
† These authors contributed equally to this work.

Recombinant expression and purification of hPL

The human pancreatic cDNA was cloned into the pTT5 vector with the N-terminal mouse IgG κ chain signal peptide and C-terminal His10-tag. Freestyle 293-F cells (Invitrogen) were cultured in SMM 293T-II medium (Sino Biological Inc.) at 37 °C, 130 rpm under 5% CO_2. The pTT5 containing human pancreatic lipase cDNA plasmid was pre-mixed with PEI MAX 40K (Polysciences) for 30 min before transfection. Transfection was started by adding plasmid-PEI MAX 40K mixture when the cell density reached 2.0×10^6 cells/mL. After 72 h, the transfected cells were centrifuged at 1000 rpm for 10 min and the conditioned medium was collected. The conditioned medium was loaded on the Ni Sepharose excel resin (GE Healthcare). The resin was washed with wash buffer 1 containing 25 mM Tris-HCl, pH 7.4, 150 mM NaCl, 1 mM $CaCl_2$, 2 mM imidazole. The protein was eluted with elute buffer 1 containing 25 mM Tris-HCl, pH 7.4, 150 mM NaCl, 1 mM $CaCl_2$, 200 mM imidazole. The eluted protein was diluted 10 times with a buffer (25 mM Tris-HCl, pH 8.0, 150 mM NaCl, 1 mM $CaCl_2$) and incubated with Ni-NTA agarose (Qiagen) for further purification. The agarose was washed with wash buffer 2 containing 25 mM Tris-HCl, pH 8.0, 150 mM NaCl, 1 mM $CaCl_2$, 20 mM imidazole. Then the protein was eluted with elute buffer 2 containing 25 mM Tris-HCl, pH 8.0, 150 mM NaCl, 1 mM $CaCl_2$, 300 mM imidazole. The human pancreatic lipase-containing fractions were concentrated using a 10 kDa molecular weight cut-off (MWCO) concentrator (Millipore). The concentrated sample was purified on a Superdex 200 10/300 GL column (GE Healthcare) equilibrated in protein storage buffer (25 mM Tris-HCl, pH 7.4, 150 mM NaCl, 1 mM $CaCl_2$).

Preparation of S9 fractions from monkey tissues and AR42J cells

Monkey pancreas samples were washed with PBS solution (0.1 M, pH=7.4) containing EDTA (1.0 mM). The weighed samples were cut into pieces in PBS solution. These fragments were ground using a fully automated sample freezer grinder (60 Hz, 60 s twice). The homogenate of the fragments was centrifuged at 9000 G for 20 min to obtain the S9 fraction. The cells were cultured in the medium containing fetal bovine serum to 80% density. The cells were broken with a cell crusher, and the supernatant (S9) was collected after 9000g centrifuge for 20min.

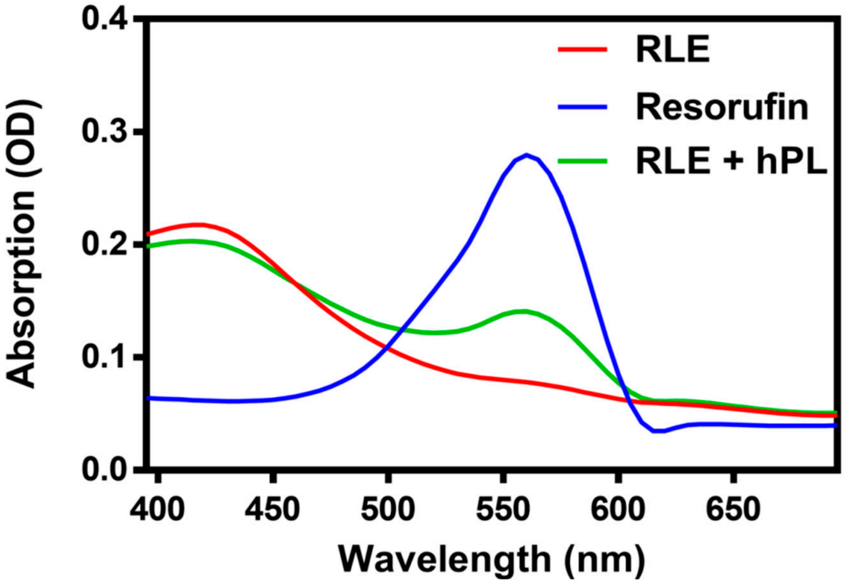

Figure S1. The change in absorption spectrum of **RLE** (20 μM) in presence of hPL (10 μg/mL).

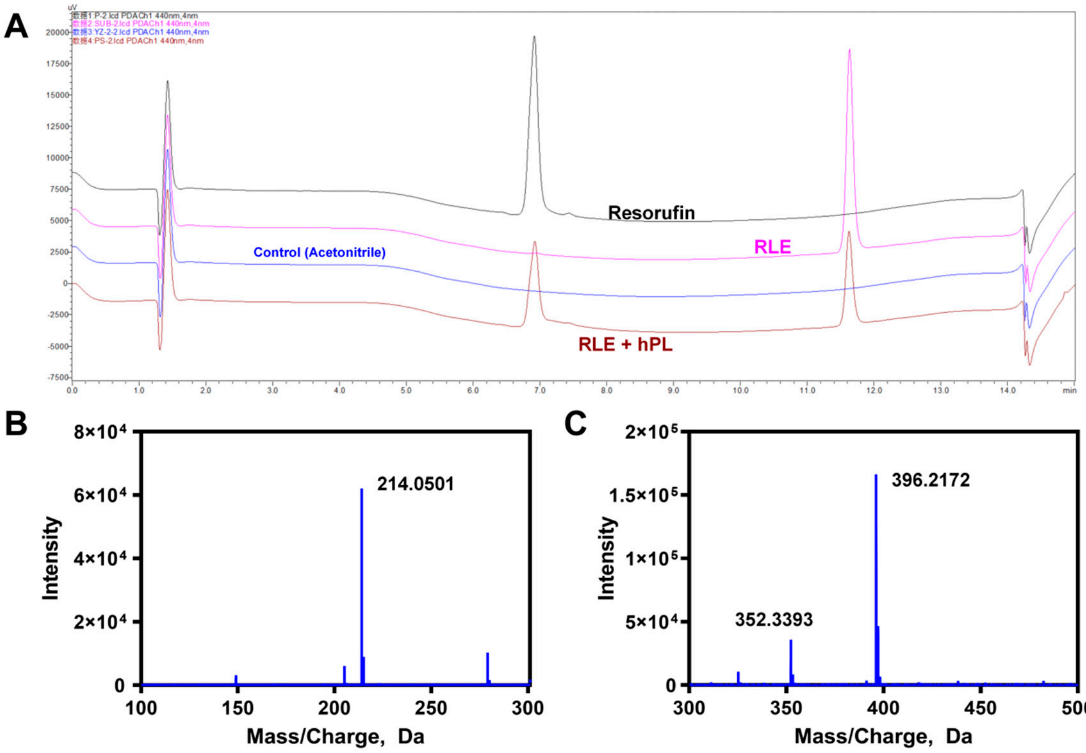

Figure S2. (A) Representative LC-UV chromatograms of **RLE** incubation samples at 37 °C, UV detector was set at 440 nm. Mass spectra of **RLE** with the quasi-molecular ion peak m/z = 396.2172 (C), and its hydrolytic product **Resorufin** with the quasi-molecular ion peak m/z = 214.0501 (B) monitored under positive mode.

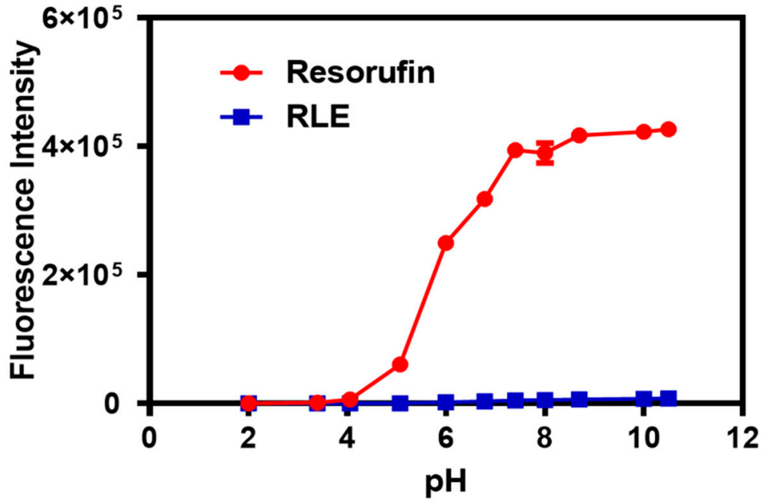

Figure S3. The effects of pH values on the fluorescence intensity of RLE and its metabolite Resorufin (5 μM), PMT Gain = 500 volts.

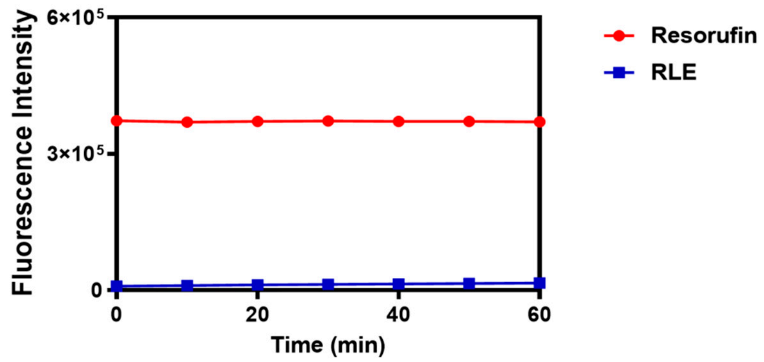

Figure S4. The photostability of RLE (5 μM) and Resorufin (5 μM), following continuous illumination at 550 nm for different time, PMT Gain = 500 volts.

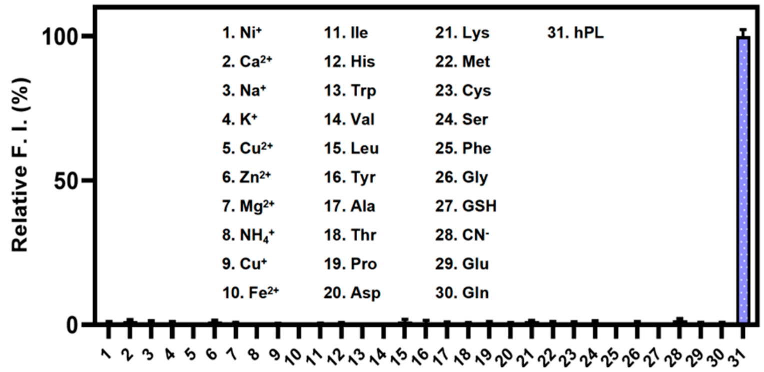

Figure S5. Fluorescence responses of **RLE** (5 μM) to various analytes in aqueous solution. Relative fluorescence intensity (%) = Fluorescence intensity of adding analytes / Fluorescence intensity of hPL.

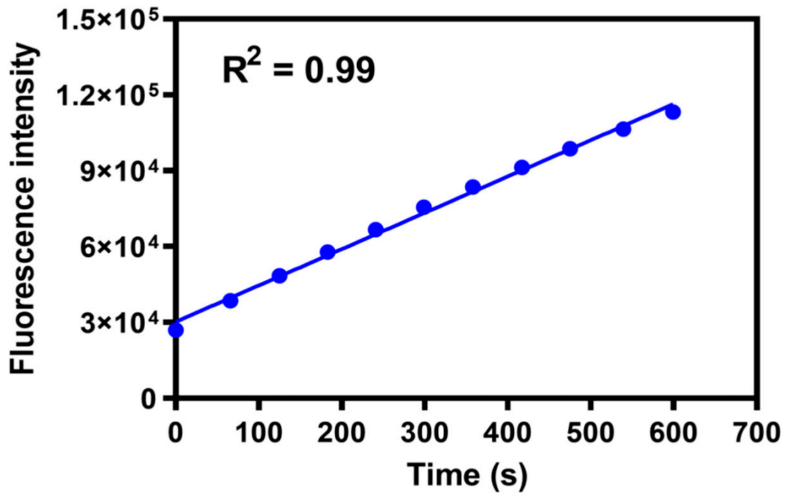

Figure S6. The Linear in fluorescence intensity of **RLE** (5 µM) over time upon addition of hPL (1 µg/mL) in buffer at 37 °C.

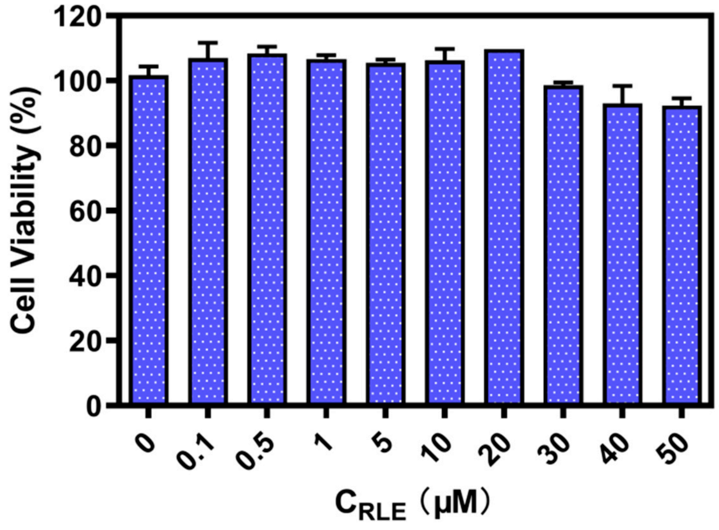

Figure S7. The cytotoxicity of **RLE** in AR42J.

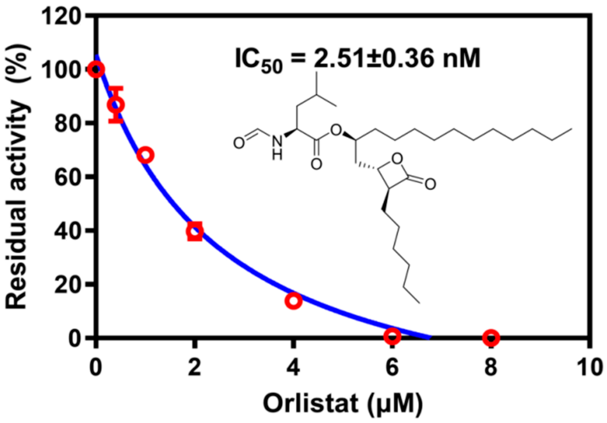

Figure S8. Dose-inhibition curves of orlistat against hPL catalysed **RLE** hydrolysis. All data were expressed as mean ± SD.

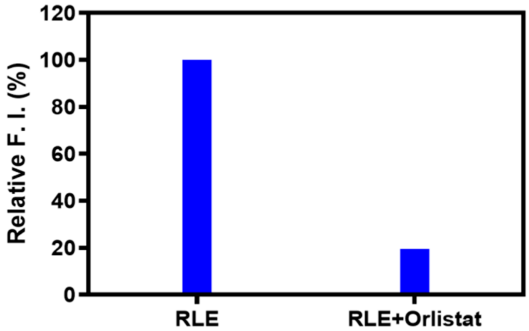

Figure S9. Graphical quantification of average fluorescence intensity of PL activities by **RLE** in AR42J cells.

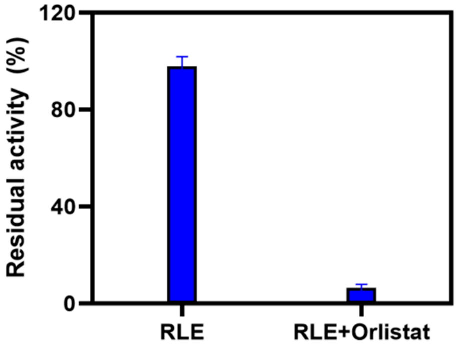

Figure S10. Inhibitory effect of orlistat (25 μM) on PL catalyzed **RLE** hydrolysis in AR42J cells S9.

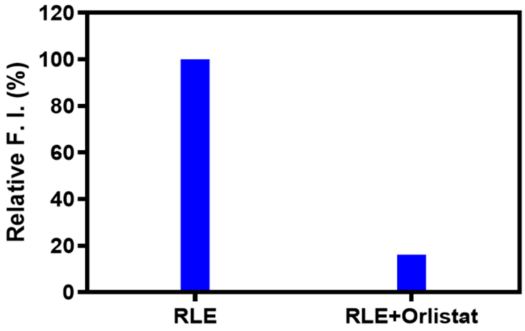

Figure S11. Graphical quantification of average fluorescence intensity of PL activities by **RLE** in Monkey pancreatic tissues.

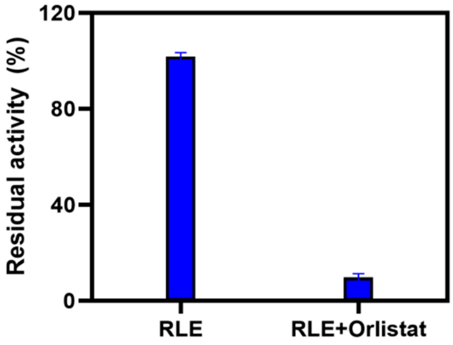

Figure S12. Inhibitory effect of orlistat (25 μM) on PL catalyzed **RLE** hydrolysis in monkey pancreas S9.

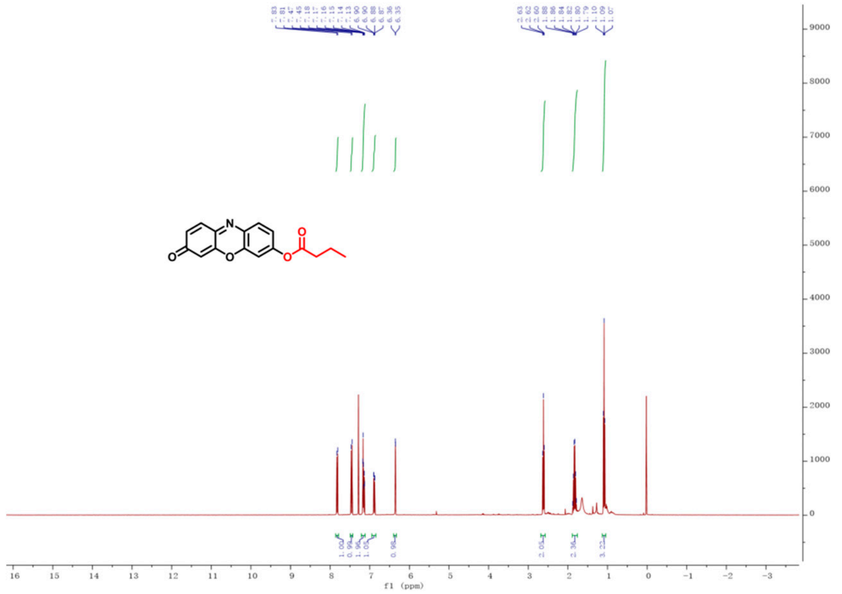

Figure S13. ^{1}H NMR spectra of compound **Resorufin butyryl ester**.

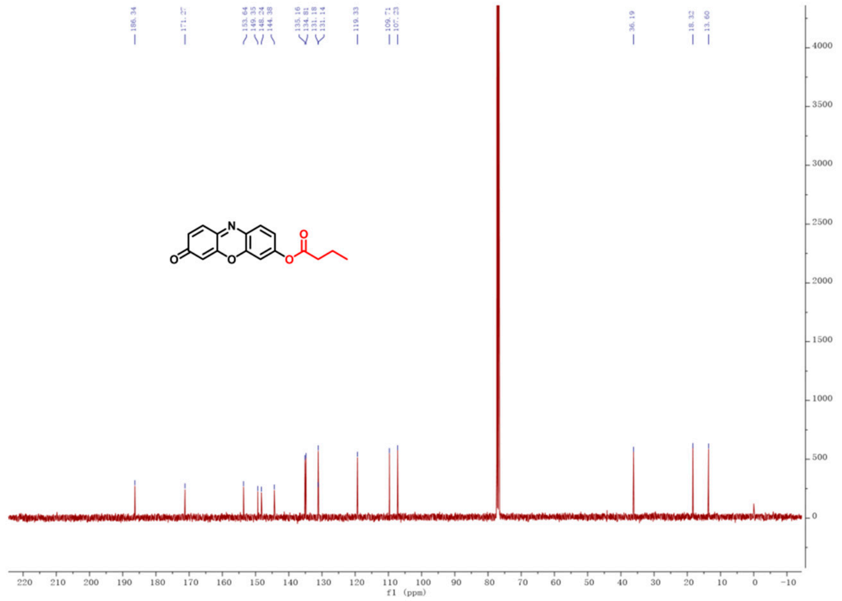

Figure S14. ^{13}C NMR spectra of compound **Resorufin butyryl ester**.

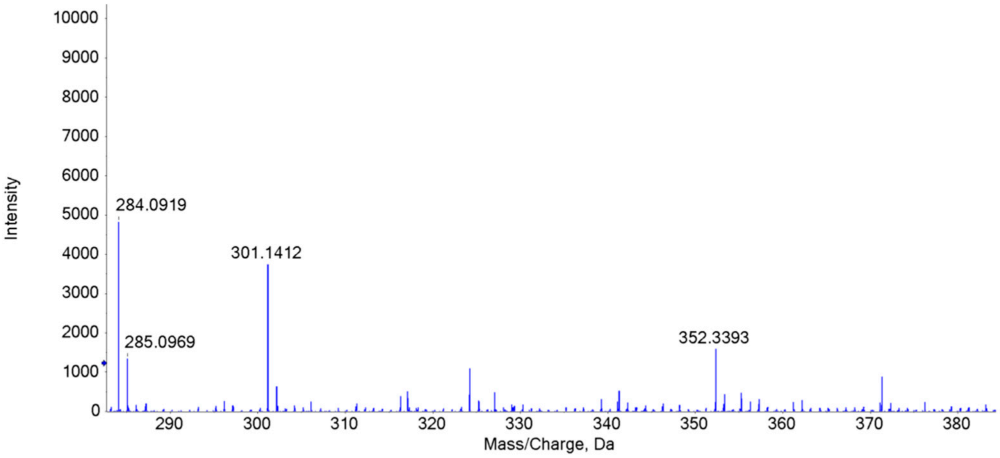

Figure S15. HRMS spectrum of compound **Resorufin butyryl ester**.

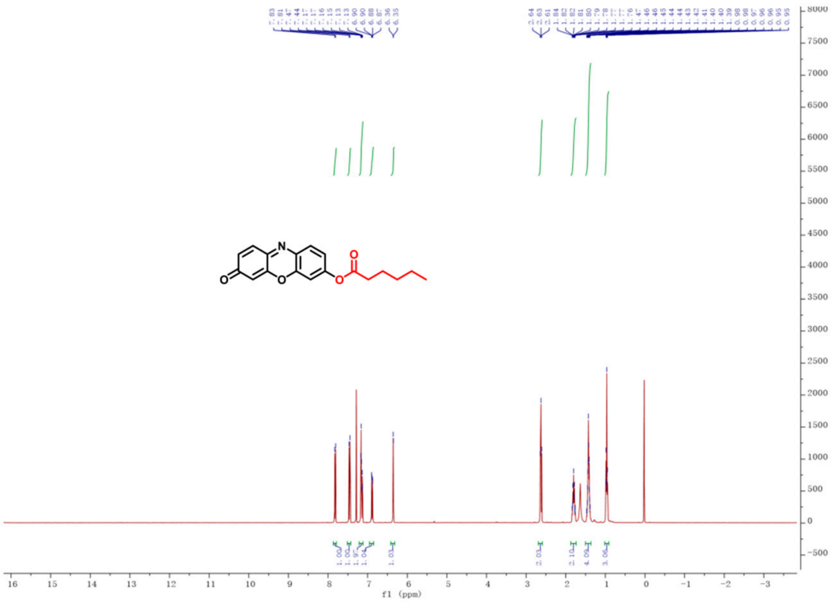

Figure S16. ^{1}H NMR spectra of compound **Resorufin hexanoyl ester**.

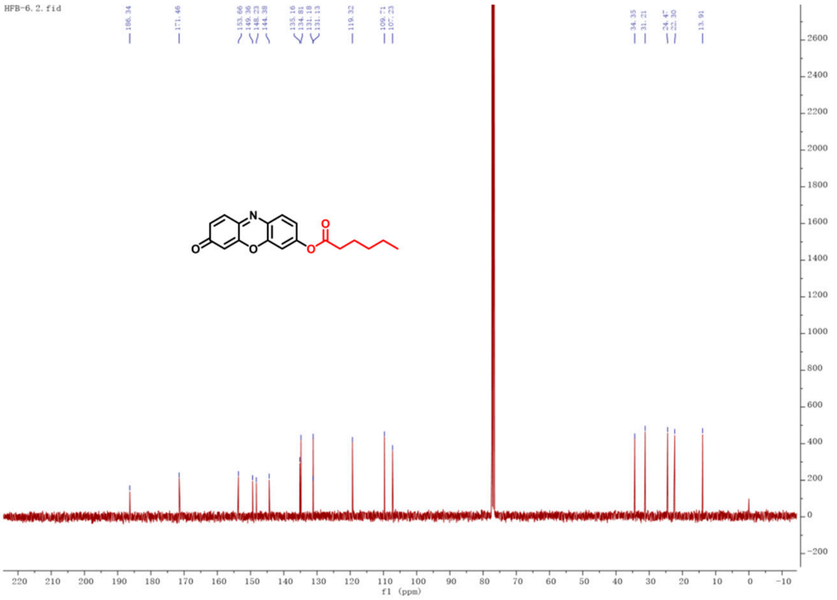

Figure S17. ^{13}C NMR spectra of compound **Resorufin hexanoyl ester**.

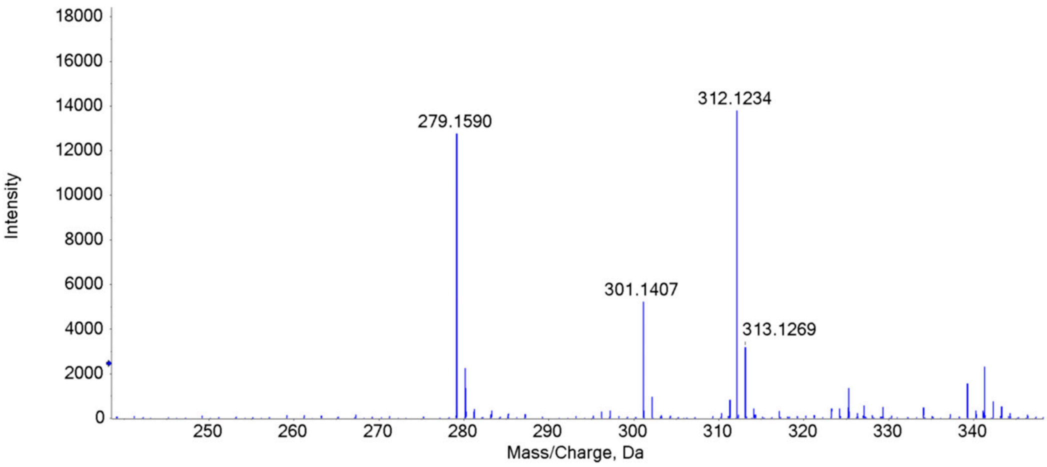

Figure S18. HRMS spectrum of compound **Resorufin hexanoyl ester**.

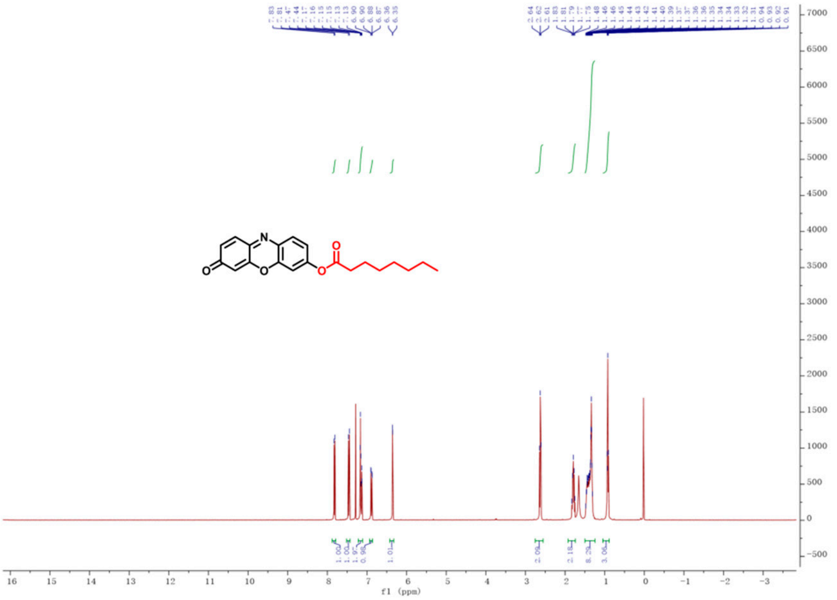

Figure S19. ^{1}H NMR spectra of compound **Resorufin octanoyl ester**.

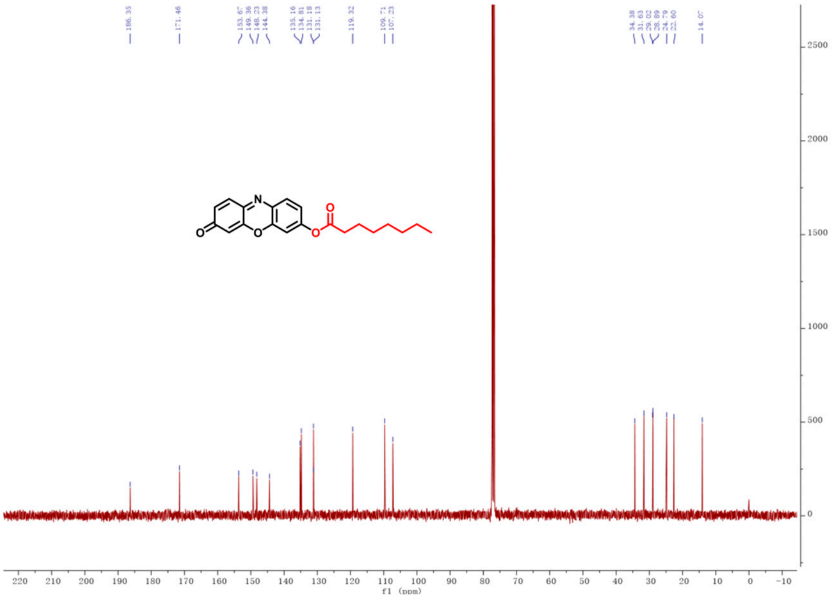

Figure S20. ^{13}C NMR spectra of compound **Resorufin octanoyl ester**.

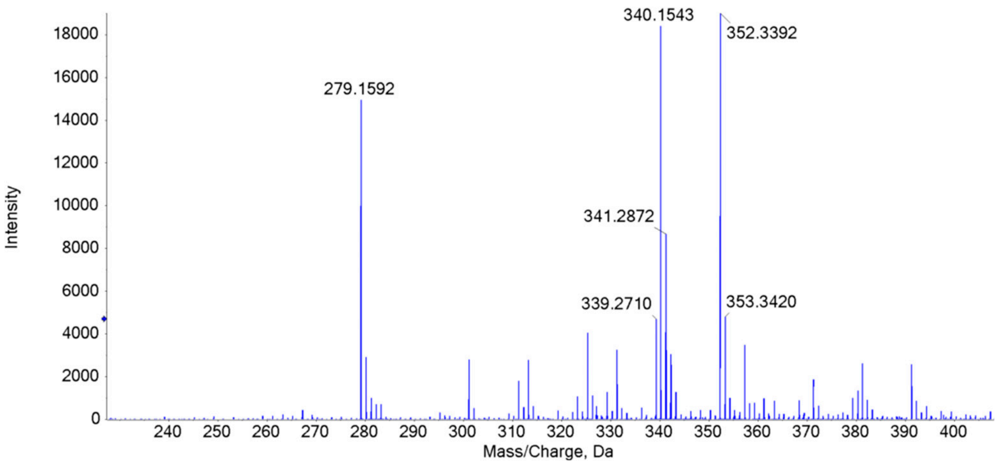

Figure S21. HRMS spectrum of compound **Resorufin octanoyl ester**.

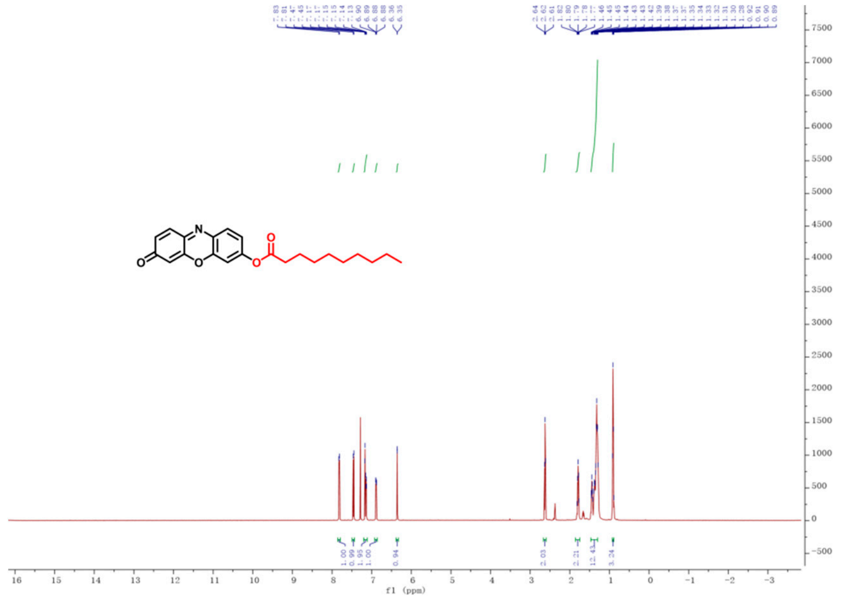

Figure S22. ^{1}H NMR spectra of compound **Resorufin decanoyl ester**.

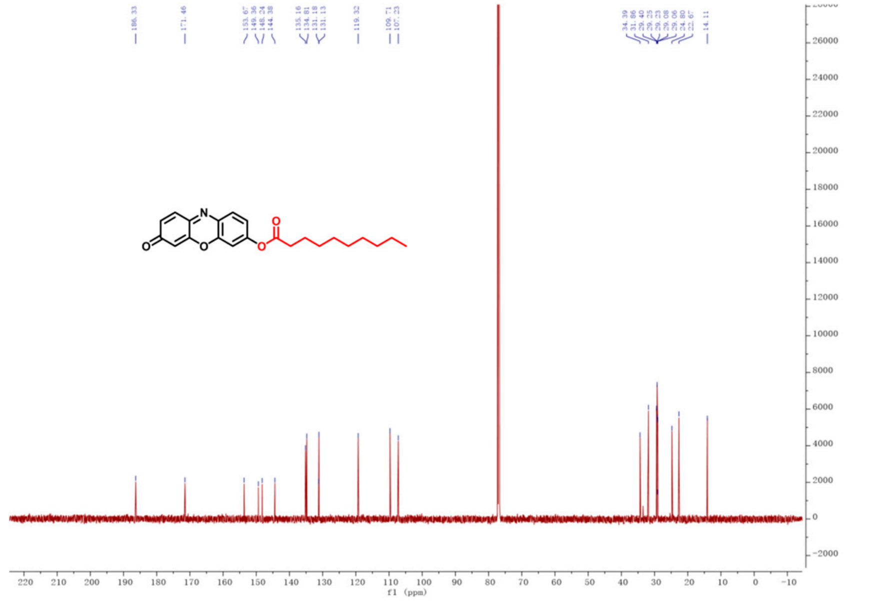

Figure S23. ^{13}C NMR spectra of compound **Resorufin decanoyl ester**.

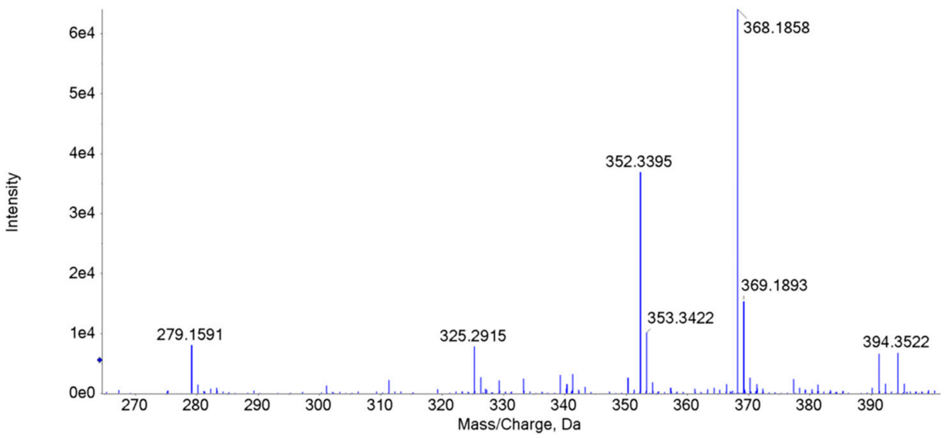

Figure S24. HRMS spectrum of compound **Resorufin decanoyl ester**.

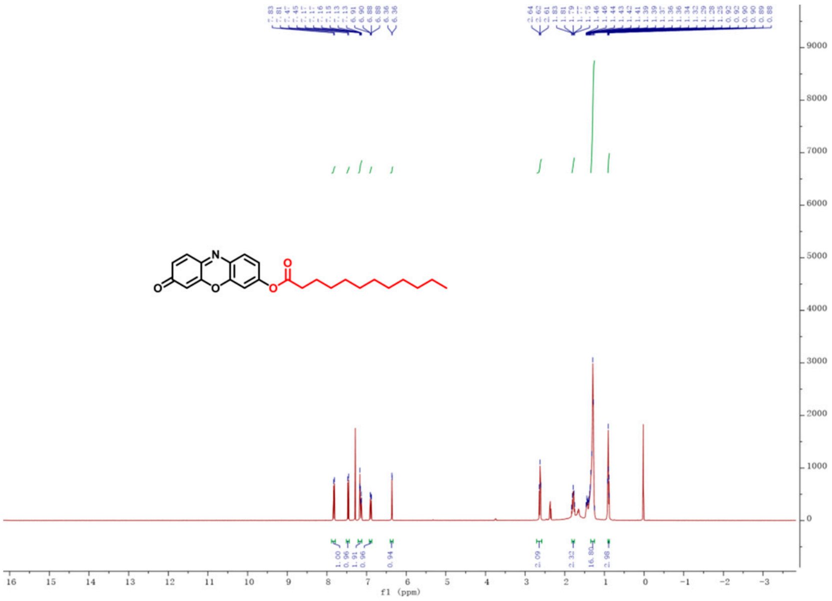

Figure S25. ^{1}H NMR spectra of compound **RLE**.

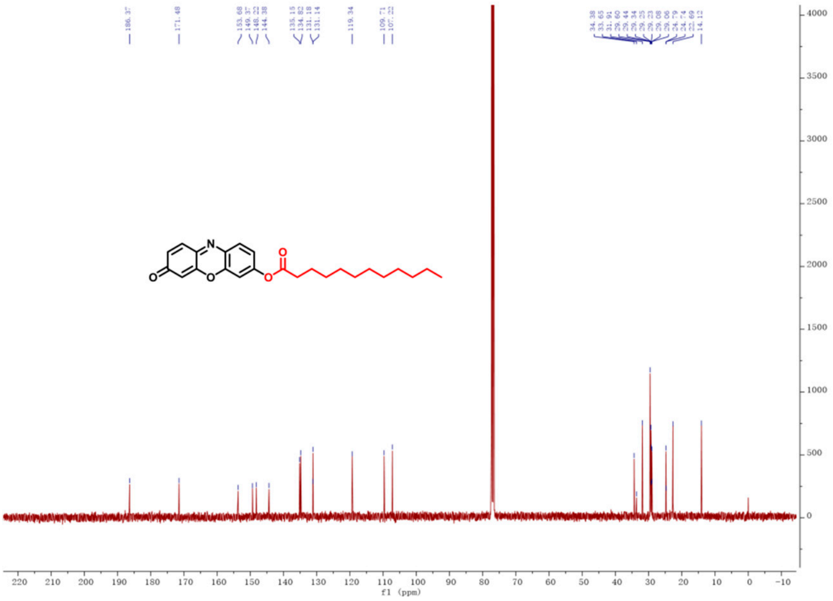

Figure S26. ^{13}C NMR spectra of compound **RLE**.

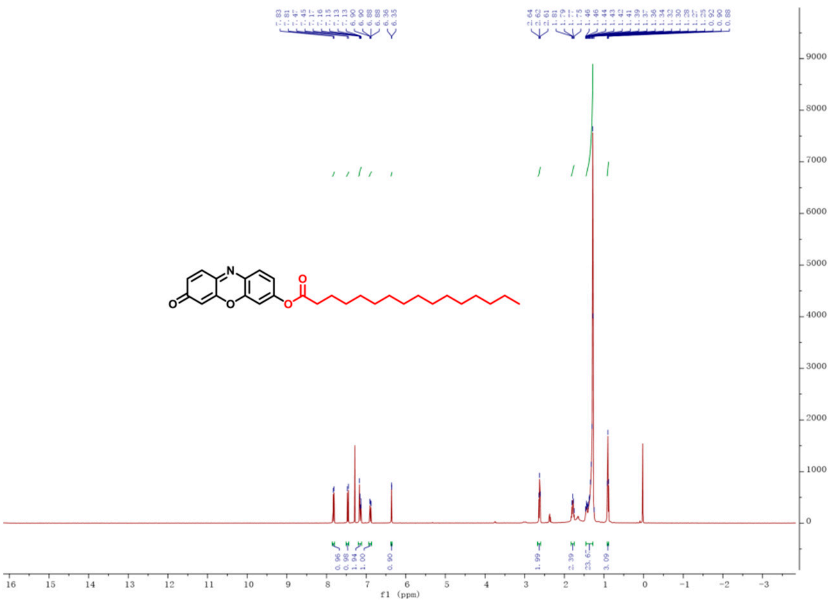

Figure S27. ^{1}H NMR spectra of compound **Resorufin palmitoyl ester**.

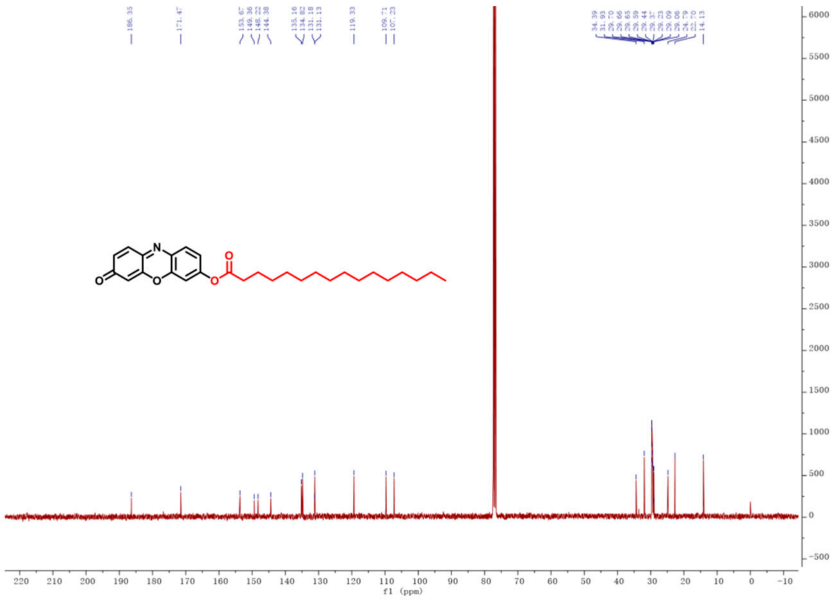

Figure S28. ^{13}C NMR spectra of compound **Resorufin palmitoyl ester**.

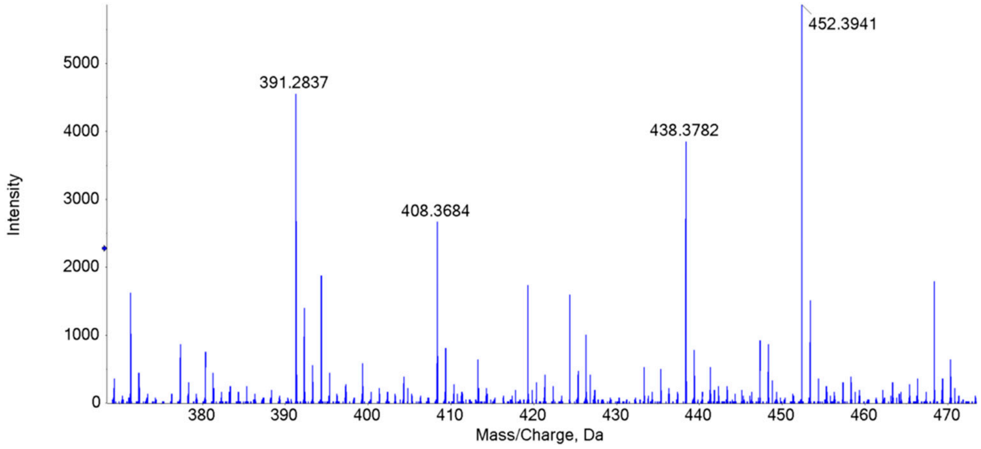

Figure S29. HRMS spectrum of compound **Resorufin palmitoyl ester**.

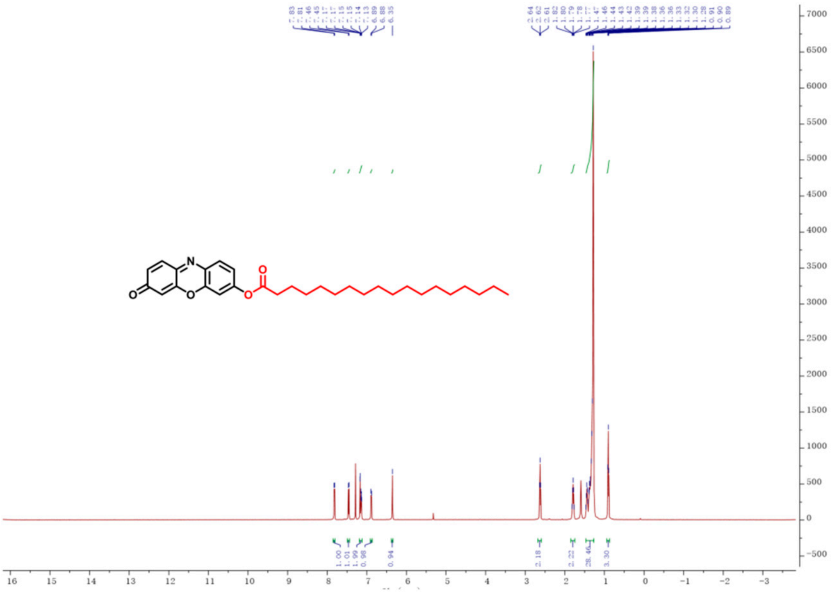

Figure S30. ^{1}H NMR spectra of compound **Resorufin stearyl esters**.

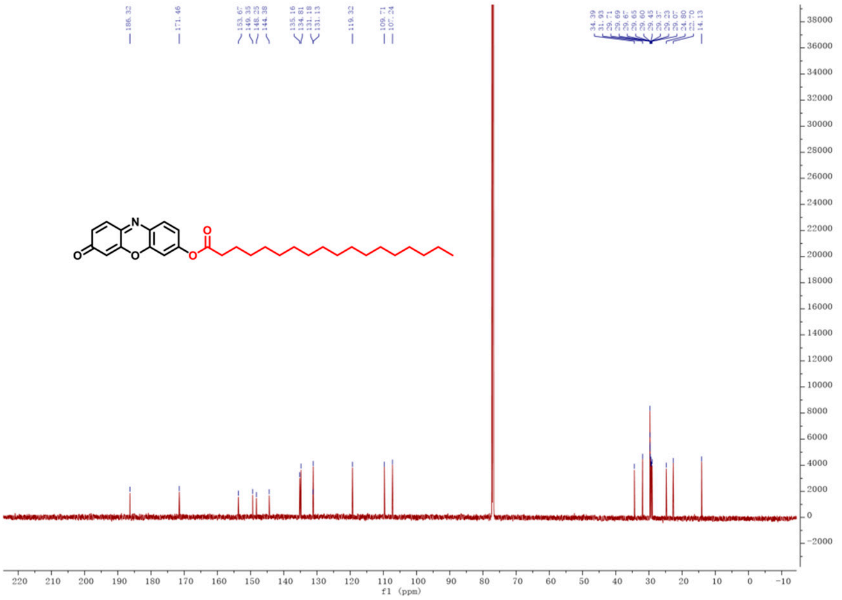

Figure S31. ^{13}C NMR spectra of compound **Resorufin stearyl esters**.

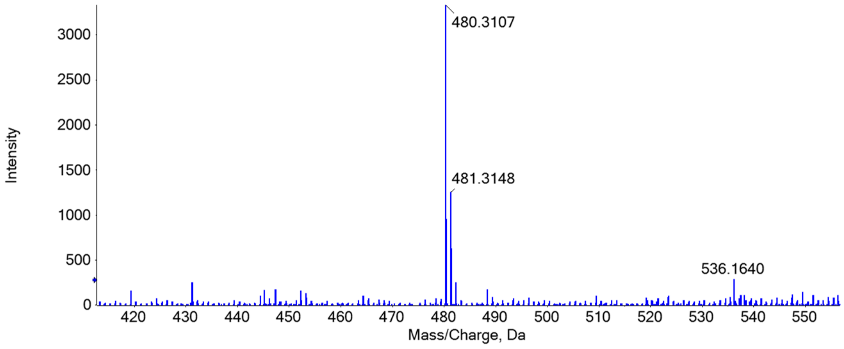

Figure S32. HRMS spectrum of compound **Resorufin stearyl esters**

Table S1. The reported fluorescent substrates for sensing pancreatic lipase.

Fluorescent substrate	Target enzyme	Detection conditions	LOD	Ref.
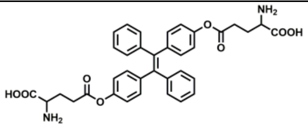	PPL	λ_{ex}=360 nm<br>λ_{em}=453 nm	0.13 U/L	[1]
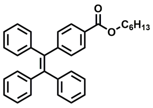	PPL	λ_{ex}=350 nm<br>λ_{em}=420 nm	0.1 mg/mL	[2]
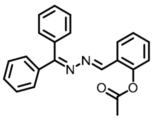	pPL	λ_{ex}=380 nm<br>λ_{em}=562 nm	0.05 U/L	[3]
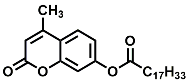	hPL	λ_{ex}=360 nm<br>λ_{em}=460 nm	N.D.	[4]
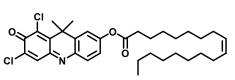	hPL	λ_{ex}=600 nm<br>λ_{em}=660 nm	0.40 μg/mL	[5]
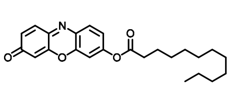	hPL	λ_{ex}=570nm<br>λ_{em}=590 nm	0.369 μg/mL	This study

Table S2. Residual activity of 94 natural products derived from herbs (10 μM, final concentration) against hPL-catalyzed **RLE** hydrolysis.

No.	Compound	MW	Residue activity (%)
a1	Ctrl (DMSO only)	-	100
a2	Orlistat (positive inhibitor)	477.04	5.33
a3	Ginkgetin	566.51	39.42
a4	Loureirin A	286.32	67.52
a5	Loureirin B	316.35	79.89
a6	Licochalcone D	354.4	80.45
a7	Licochalcone B	286.28	58.04
a8	Isorhynchophylline	384.47	87.99
a9	Apigenin	270.24	102.46
a10	Palmatine chloride	387.86	87.01
a11	Wogonin	284.26	106.17
a12	Genistein	270.24	106.48
b1	Dehydrocorydaline	366.436	123.69

b2	Coptisine chloride	355.77	113.58
b3	Catharanthine	336.43	81.11
b4	Liquiritin	418.4	105.77
b5	Formononetin	268.26	104.25
b6	Berbamine Hydrochloride	665.65	69.86
b7	Khasianine	721.93	102.50
b8	Pimpinellin	246.22	61.25
b9	Oxysophocarpine	262.35	98.58
b10	Obacunon	454.51	79.60
b11	Ginkgolide C	440.4	106.22
b12	Ginkgolide B	424.4	120.07
c1	Ginkgolide A	408.4	125.97
c2	Baicalein	Baicalein	48.80
c3	Alantolactone	232.32	61.11
c4	Isoalantolactone	232.32	70.02
c5	Andrographolide	350.45	70.38
c6	Myricitrin	464.38	91.12
c7	Dihydromyricetin	320.25	95.62
c8	TGaxifolin	304.25	97.17
c9	*p*-Coumaric acid	164.16	98.98
c10	Dihydrokaempferol	288.25	109.31
c11	Sinomenine	329.39	101.42
c12	Tiapride	328.43	108.20
d1	Dehydroandrographolide	332.43	89.55
d2	Esculetin	Esculetin	86.12
d3	Quercetin	302.24	47.29
d4	Celastrol	450.61	84.16
d5	Hydroxysafflor yellow A	612.54	76.59
d6	Orientin	448.38	76.06
d7	Bergapten	216.19	54.11
d8	Isoimperatorin	270.28	72.90
d9	Curcumin	368.38	75.83
d10	Praeruptorin E	428.47	87.81
d11	Imperatorin	270.28	75.61
d12	8-Methoxypsoralen	216.19	90.65
e1	trans-Cinnamic acid	148.16	72.50
e2	Salvigenin	328.32	75.70
e3	Myricetin	318.24	56.43
e4	Herbacetin	302.24	83.59
e5	Oridonin	364.44	66.82

e6	Limonin	470.51	85.30
e7	Benzyl cinnamate	238.28	74.62
e8	Coptsine	320.32	106.42
e9	Irisflorentin	386.35	66.38
e10	Coptisine	320.32	101.77
e11	4H-1-Benzopyran-4-one, 3-[[6-*O*-(6-deoxy-α-L-mannopyranosyl)-*β*-D-glucopyranosyl]oxy]-5,7-dihydroxy-2-(3,4,5-trihydroxyphenyl)	626.52	116.73
e12	Astragalin	448.38	107.88
f1	Nobiletin	402.39	80.44
f2	Homoharringtonine	545.63	74.77
f3	Z-Ligustilide	190.24	83.60
f4	3-N-butyl-4,5-dihydrophthalide	192.25	75.65
f5	Isorhamnetin-3-glucoside	478.4	93.93
f6	Xanthohumol	354.4	81.19
f7	Phloretin	274.27	81.23
f8	Xanthohumol L	370.4	100.78
f9	Phlorizin dihydrate	436.41	85.09
f10	Berberrubine	357.79	86.09
f11	Shikonin	288.31	107.55
f12	Toosendanin	574.62	72.37
g1	Methyl caffeate	194.18	72.74
g2	2-(3,4-Dihydroxyphenyl)-3,6,7-trihydroxy-5-methoxy-4H-1-benzopyran-4-one	332.26	59.87
g3	Caffeic acid	180.16	71.45
g4	Sesamol	138.12	103.95
g5	phytolaccagenin	532.70	85.42
g6	Pinocembrin	256.25	91.25
g7	Isovitexin	432.38	79.86
g8	Methylophiopogonanone A	342.34	80.44
g9	Isoginkgetin	566.51	54.78
g10	Ipriflavone	280.32	84.50
g11	Timosaponin A-III	740.92	95.12
g12	Salvianolic acid	494.45	96.06
h1	Lysionotin	344.31	84.30
h2	Calycosin 7-*O*-glucoside	446.41	84.02
h3	Cassiaside	404.36	81.67
h4	Platycodin D	1225.34	89.14
h5	Alpinetin	270.28	107.78
h6	Glycitin	446.41	99.85

h7	Epmedin C	822.8	81.94
**h8**	**Procyanidin**	**594.52**	**5.72**
**h9**	**Carnosol**	**330.42**	**4.20**
**h10**	**Sciadopitysin**	**580.54**	**5.25**
h11	5,7-Dihydrox -4'-methoxyisoflavone	284.26	100.90
h12	Morin hydrate	320.25	96.10

Table S3. Residual activity of 94 drugs (10 μM, final concentration) against hPL-catalyzed **RLE** hydrolysis.

No.	Compound	MW	Residue activity (%)
a1	Ctrl (DMSO only)	-	100
a2	Orlistat (positive inhibitor)	477.04	5.31
a3	Etoposide	588.56	65.05
a4	Crizotinib	450.34	85.14
a5	Digoxin	780.95	99.51
a6	Quinidine	324.42	100.77
a7	Boldenone	286.41	66.33
a8	Omeprazole	345.42	94.84
a9	Clopidogrel sulfate	405.87	90.49
a10	Celecoxib	381.37	90.67
a11	diclofenzc	296.10	97.62
a12	Eslicarbazepine	254.28	103.44
b1	Gemcitabine	263.20	105.06
b2	Glimepiride	490.62	90.05
b3	Prednisolone	360.45	82.34
b4	Lamotrigine	256.09	84.88
b5	Amlodipine	408.88	85.85
b6	Nimodipine	418.44	98.69
b7	Sulbactam sodium	257.24	88.89
**b8**	**Ivermectin**	**875.09**	**3.05**
b9	Cloxacillin benzathine	676.23	72.17
b10	Nisoldipine	388.41	75.08
b11	Unii-33Y9anm545	473.99	96.93
b12	Methylprednisolone	374.47	44.82
c1	Megestrol acetate	384.51	44.82
c2	Felodipine	384.25	93.02
c3	Indometacin	357.79	102.95
c4	Clozapine	326.82	97.86

c5	Lovastatin	404.54	80.96
c6	Loratadine	382.88	96.14
c7	Midazolam	325.77	96.73
c8	Metoprolol tartrate	684.82	135.23
c9	Furosemide	330.74	100.00
c10	Probenecid	285.36	93.75
c11	Metronidazole	171.15	78.49
c12	Sorafenib tosylate	637.03	56.84
**d1**	**Raloxifene**	**473.58**	**4.05**
d2	Zidovudine	283.24	73.04
d3	Dasatinib	488.01	91.97
d4	Reserpine	608.69	94.24
d5	Warfarin sodium	330.31	84.05
d6	Simvastatin	418.57	84.18
d7	Atazanavir	704.86	79.92
d8	Diphenhydramine Hydrochloride	291.82	88.58
d9	3-Hydroxytyramine hydrochloride	189.64	86.11
d10	Terbinafine Hydrochloride	327.89	91.01
d11	CeftazidiMe	546.58	118.41
d12	Alprazolam	308.76	68.13
e1	Carbamazepine	236.27	104.15
e2	Oseltamivir phosphate	410.40	101.77
e3	Linagliptin	472.54	108.93
e4	Ibuprofen	206.28	103.68
e5	Gestrinone	308.41	66.72
e6	Nitrendipine	360.36	92.86
e7	Vandetanib	475.35	104.95
e8	Naproxen	230.26	116.02
e9	bupropion hydrochloride	276.20	120.79
e10	AZD-9291	499.61	92.69
e11	Cyclopropyl 2-fluorobenzyl ketone	178.20	117.10
e12	Benorilate	313.30	76.99
**f1**	**Sorafenib tosylate**	**637.03**	**44.04**
f2	Amikacin	585.60	73.77
f3	Diflunisal	250.20	118.03
f4	Donepezil Hydrochloride	415.95	100.82
f5	Oxaprozin	293.32	81.83
f6	Hydrocortisone	362.47	56.27
f7	Cephalexin	347.39	109.58
f8	Ofloxacin	361.37	87.95

f9	Etonogestrel	324.46	62.97
f10	Irinotecan	586.68	105.25
f11	Dextromethorphan	271.4	89.61
f12	Terbutaline	225.28	108.89
g1	Cimetidine	252.34	111.11
g2	Ritonavir	720.94	97.04
g3	Menthofuran	150.22	108.33
g4	Rivaroxaban	435.88	118.66
g5	Polymyxin B	1203.5	52.81
g6	Imipramine hydrochloride	316.87	92.51
g7	Fenofibrate	360.83	94.68
g8	Medroxyprogesterone Acetate	386.52	68.77
g9	Fenoprofen	242.27	91.13
g10	Amiloride hydrochloride	266.09	109.84
g11	Vancomycin	1449.25	96.04
g12	Ethionamide	166.24	91.71
h1	Sulfamethoxazole	253.28	98.83
h2	Hydrochlorothiazide	297.74	99.42
h3	Ezetimibe	409.43	119.27
h4	Linezolid	337.35	91.81
h5	Tenofovir disoproxil	519.44	91.86
h6	4-Cholesten-3-one	384.64	77.59
h7	Bambuterol hydrochloride	403.90	93.23
h8	Cyclophosphamide	261.09	91.59
h9	Entinostat	376.41	102.02
h10	Testosterone propionate	344.50	65.37
h11	Genipin	226.23	99.28
h12	Remdesivir	602.58	98.67

References

[1] Shi, J., Deng, Q., Wan, C., Zheng, M., Huang, F., & Tang, B. (2017). Fluorometric probing of the lipase level as acute pancreatitis biomarkers based on interfacially controlled aggregation-induced emission (AIE). *Chemical science*, *8*(9), 6188–6195.

[2] Shi J., Zhang S., Zhen M. M., Deng Q. C., Zheng C., Jing L., Huang F. H. (2017). A novel fluorometric turn-on assay for lipase activity based on an aggregation-induced emission (AIE) luminogen. *Sens. Actuator B Chem.* 238 765-771.

[3] Guan, P., Liu, Y., Yang, B., Wu, Y., Chai, J., Wen, G., & Liu, B. (2021). Fluorometric probe for the lipase level: Design, mechanism and biological imaging application. *Talanta*, *225*, 121948.

[4] Ivanov Sergey A., Nomura K., Malfanov Ilya L., Sklyar Ilya V., Ptitsyn Leonid R. (2011). Isolation of a novel catechin from Bergenia rhizomes that has pronounced lipase-inhibiting and antioxidative properties. *Fitoterapia*, 82(2), 212-8.
[5] Hu, Q., Tian, Z., Wang, H., Huang, J., Wang, F., Zhao, B., He, R., Jin, Q., Hou, X., Hou, J., Fang, S., Wang, P., & Ge, G. (2021). Rational design and development of a novel and highly specific near-infrared fluorogenic substrate for sensing and imaging of human pancreatic lipase in living systems. *Sensors and Actuators B-chemical*, 341, 130033.
